# The Relationship Between the Network of Non-coding RNAs-Molecular Targets and N6-Methyladenosine Modification in Colorectal Cancer

**DOI:** 10.3389/fcell.2021.772542

**Published:** 2021-12-06

**Authors:** Senxu Lu, Xiangyu Ding, Yuanhe Wang, Xiaoyun Hu, Tong Sun, Minjie Wei, Xiaobin Wang, Huizhe Wu

**Affiliations:** ^1^ Department of Pharmacology, School of Pharmacy, China Medical University, Shenyang, China; ^2^ Liaoning Key Laboratory of Molecular Targeted Anti-tumor Drug Development and Evaluation, Liaoning Cancer Immune Peptide Drug Engineering Technology Research Center, Key Laboratory of Precision Diagnosis and Treatment of Gastrointestinal Tumors, Ministry of Education, China Medical University, Shenyang, China; ^3^ Department of Medical Oncology, Cancer Hospital of China Medical University, Shenyang, China; ^4^ Shenyang Kangwei Medical Laboratory Analysis Co. Ltd., Liaoning, China; ^5^ Center of Reproductive Medicine, Shengjing Hospital of China Medical University, Shenyang, China

**Keywords:** long non-coding RNA, micro RNA, interaction network, colorectal cancer, N6-methyladenosine modification

## Abstract

Recent accumulating researches implicate that non-coding RNAs (ncRNAs) including microRNA (miRNA), circular RNA (circRNA), and long non-coding RNA (lncRNAs) play crucial roles in colorectal cancer (CRC) initiation and development. Notably, N6-methyladenosine (m^6^A) methylation, the critical posttranscriptional modulators, exerts various functions in ncRNA metabolism such as stability and degradation. However, the interaction regulation network among ncRNAs and the interplay with m^6^A-related regulators has not been well documented, particularly in CRC. Here, we summarize the interaction networks and sub-networks of ncRNAs in CRC based on a data-driven approach from the publications (IF > 6) in the last quinquennium (2016–2021). Further, we extend the regulatory pattern between the core m^6^A regulators and m^6^A-related ncRNAs in the context of CRC metastasis and progression. Thus, our review will highlight the clinical potential of ncRNAs and m^6^A modifiers as promising biomarkers and therapeutic targets for improving the diagnostic precision and treatment of CRC.

## Introduction

### Background

Colorectal cancer (CRC) remains the third most common tumor worldwide with increasing incidence and mortality rates annually. The etiology of CRC is complicated and involves a variety of risk factors such as environmental exposure, genetic alterations as well as a variety of epigenetic modifications based on global molecular biomarkers such as mRNA, microRNA (miRNA), long non-coding RNA (lncRNA), circular RNA (circRNA), etc. Genomic studies show that human ncRNA transcripts that do not encode for proteins account for approximate 98% of the total human transcripts, which consist mainly of lncRNA, miRNA and circRNA, etc. Among them, lncRNAs are non-coding RNAs longer than 200 nt, which play critical roles in regulating gene expression and chromatin dynamics (Bhan and Mandal, 2015). MiRNAs are ncRNAs with a length of 17–25 nt, which usually recognize the 3′UTR of mRNA and inhibit gene expression (Lee and Dutta, 2009). CircRNAs are single-stranded ncRNAs with a covalent closed loop structure, which play important biological functions by acting as miRNA inhibitors, protein “bait” or by encoding small peptides (Li et al., 2020). Notably, accumulating evidence shows that the dysregulated ncRNAs (such as lncRNAs, microRNAs, circRNAs, etc.) are involved in the pathological process of a variety of tumors such as prostate cancer, breast cancer, hepatic cancer, and CRC. Although several studies show that ncRNAs play critical regulatory roles in CRC by targeting different protein-coding transcripts or other ncRNAs to activate various signal pathways. However, the specific mechanism underlying the functions of ncRNAs in CRC remain unclear.

**FIGURE 1:**
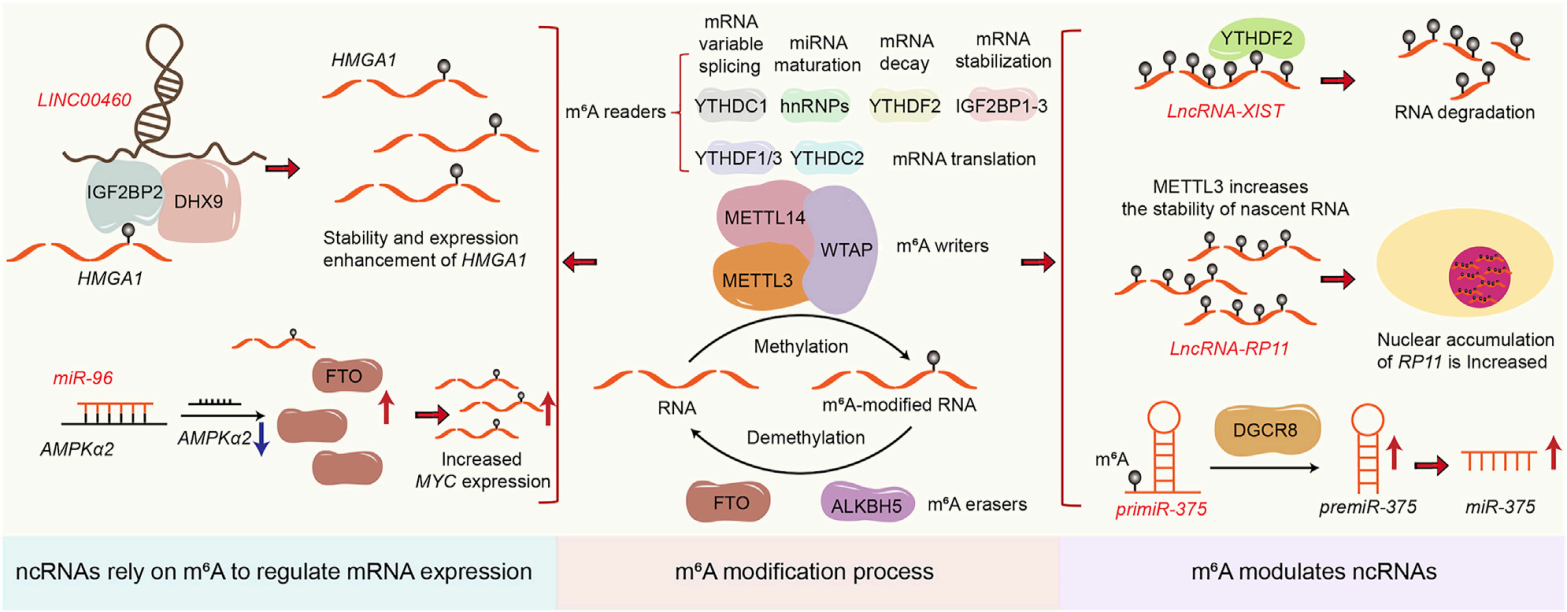
The correlation between ncRNAs and m^6^A modification. The figure shows how m^6^A regulates ncRNAs expression and how ncRNAs rely on m^6^A to regulate mRNA expression.

Accumulating researches show that ncRNAs are abnormally expressed in tissues, cells, exosomes, and blood of CRC patients (Barbagallo et al., 2018). These are identified as oncogenes or tumor suppressors that mediate CRC occurrence, metastasis, and resistance to radiotherapy and chemotherapy (Wang et al., 2017a; Luan et al., 2020; Meng et al., 2020). Although the regulatory mechanism of the biogenesis and function of ncRNAs remain unclear, existing studies show that ncRNAs play essential roles during tumorigenesis and progression through diverse mechanisms including action as miRNA sponges or baits, interaction with RNA binding proteins, translation to functional peptides as well as epigenetic modification mediated mechanisms (Ren et al., 2018a; Ni et al., 2019; Long et al., 2021). Notably, epigenetic modification of ncRNAs is a significant factor in the occurrence and development of CRC. Meanwhile, ncRNAs can also rely on epigenetic modification to regulate the expression of mRNA or ncRNAs and ultimately promote the progression of CRC. Among all epigenetic modifications, m^6^A, as a research hotspot in recent years, exerted its critical functions in the progression and development of CRC. Specifically, the m^6^A writers (METTL3, METTL14, WTAP, and other writers such as RBM15, VIRMA, CBLL1, ZC3H13) are responsible for “writing” m^6^A modification. The m^6^A erasers (FTO and ALKBH5) are responsible for “erasing” m^6^A modification. Meanwhile, m^6^A readers (YTHDC1-2, YTHDF1-3, IGF2BP1-3, HNRNPC and HNRNPA2B1) are responsible for “reading” m^6^A modification. The writers, erasers and readers of m^6^A modification can collaborate and directly participate in the progression of various types of tumors. In CRC, m^6^A modification promotes CRC angiogenesis, metastasis, and chemical resistance by regulating lncRNA stability and degradation, miRNA biogenesis, and circRNA reverse splicing and translation (Dang et al., 2021; Liu et al., 2021; Xu et al., 2021). Recently, publications focused on that m^6^A associated was supposed to regulate the expression of ncRNAs (Wu et al., 2019a; Chen et al., 2020a; Yang et al., 2020a). Yang et al. illustrated that knockdown of METTL14 enhanced the expression of long non-coding RNA XIST through YTHDF2 pathway (Yang et al., 2020a). Wu et al. clarified that m^6^A-induced lncRNA RP11 triggered the metastasis of CRC cells through the post-translational up-regulation of Zeb1 (Wu et al., 2019a). Similarly, Peng et al. demonstrated that METTL14 promoted the expression of miR-375 in an m^6^A-dependent pathway to promote the progression of CRC (Chen et al., 2020a). Furthermore, not only m^6^A can regulate the expression of ncRNAs, ncRNAs are capable to regulate the m^6^A level of RNA as well. For example, miR-96 downregulated AMPKα2, thereby blocking its m^6^A modification and leading to increased FTO expression and subsequent upregulation of MYC expression (Yue et al., 2020); LNC00460 directly interacted with IGF2BP2 and DHX9 to bind to the 3′UTR of HMGA1 mRNA, thereby increasing the stability of HMGA1 mRNA (Hou et al., 2021); m^6^A modified circNSUN2 stabilized HMGA2 mRNA and ultimately promoted liver metastasis of CRC by forming a circNSUN2/IGF2BP2/HMGA2 RNA-protein ternary complex. Thus, linking ncRNAs and m^6^A modifications is essential for advancing future diagnostic and therapeutic inventions (Chen et al., 2019a). The correlation between ncRNAs and m^6^A modification is shown in Figure 1.

**FIGURE 2:**
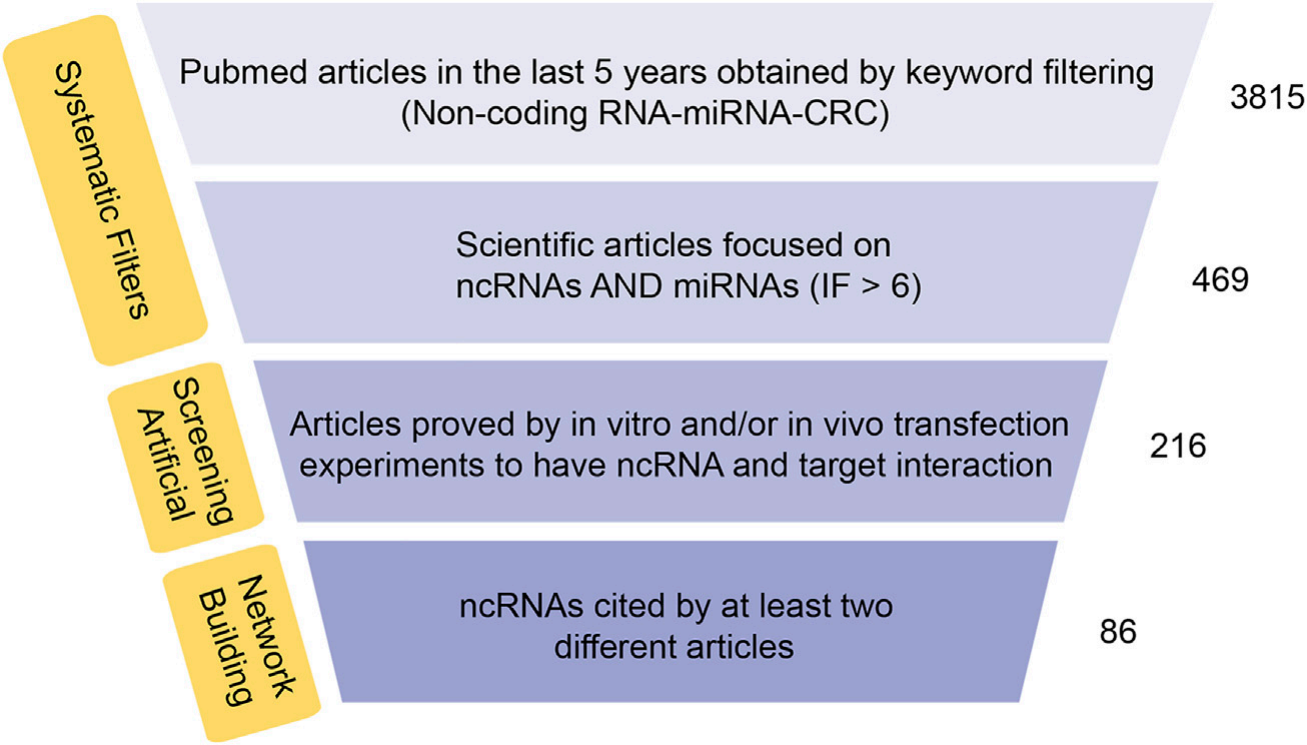
Synthesis of data-approach used to build the network ncRNAs-target. Flow chart of RNA network construction.

**FIGURE 3:**
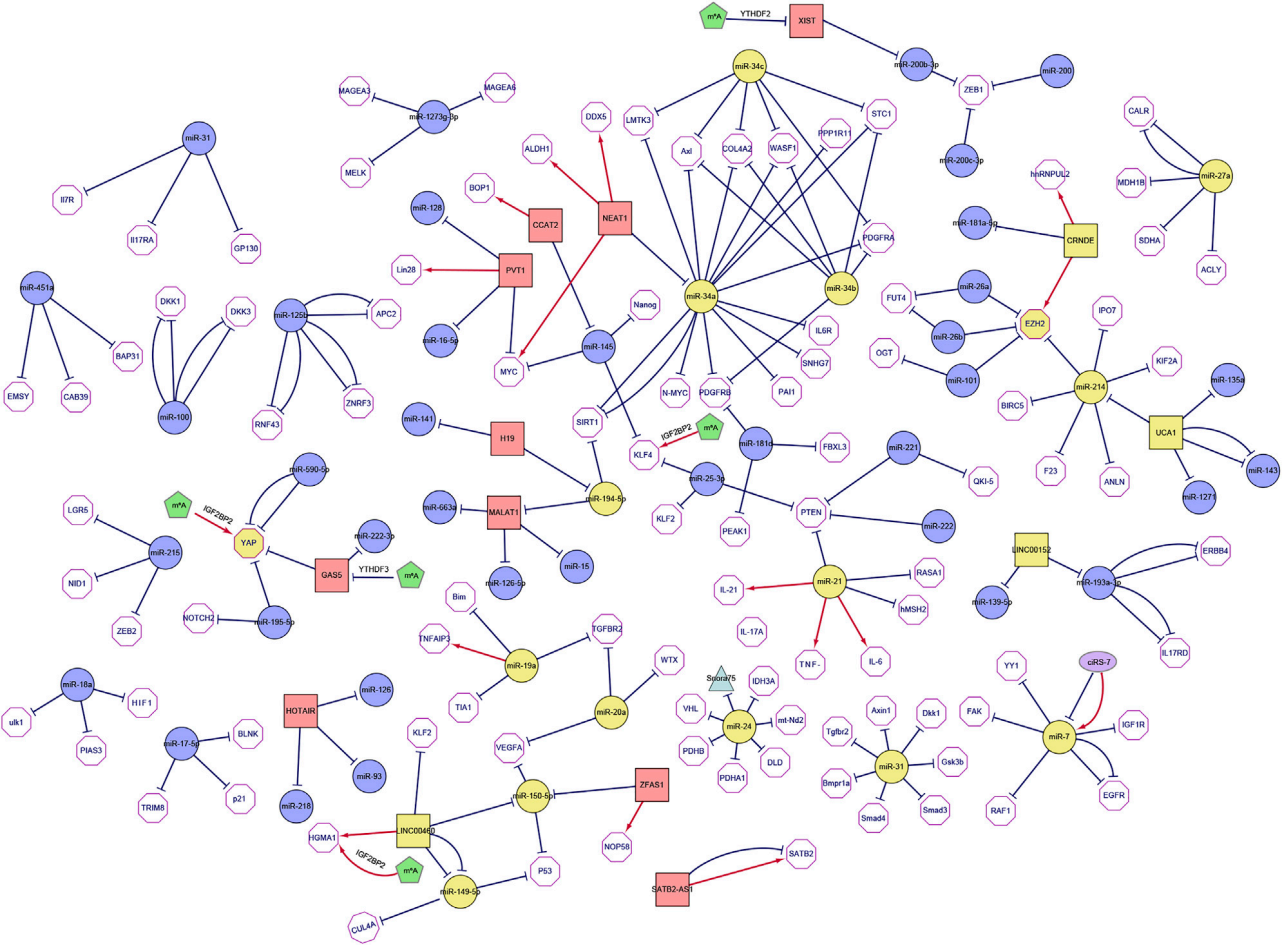
The network of non-coding RNAs and its targets in colorectal cancer. The figure shows ncRNAs reported in at least two different literature sources (the squares represent long non-coding RNAs, the purple ellipses represent circular RNAs, the circles represent microRNAs, the triangles represent snoRNAs, the pentagon represents m^6^A modification and yellow indicates core genes). The target mRNA of ncRNA is represented by a hexagon, and each target is reported in PUBMED. When the interaction is described in multiple articles, multiple line segments are drawn between the two nodes. The edges are directed (i.e., from the non-coding RNA to its target which could either be coding or non-coding). In the figure, red regular arrows indicate active links, and black flat arrows indicate inhibited links.

The current researches on ncRNAs in CRC are limited on the selection of one or more representative ncRNAs in clinical genomics. In these studies, the CRC transcriptome is analyzed in cohort retrospectively and usually lacks a holistic approach. The current research is primarily based on miRNA as the representative of ncRNAs, which usually regulate biological functions and promote or inhibit the occurrence of tumors by affecting the expression of multiple direct or indirect targets in common biological networks. For each ncRNA, hundreds of mRNAs or other ncRNAs are generally enriched as direct or indirect targets, and the coordination of many of these can be regulated to produce a series of biological consequences. Using this functional feature to our advantage, we took a data-driven approach and collected all the articles on CRC-related ncRNAs and miRNAs from PubMed in the last 5 years, and set IF > as the threshold. Next, we combined text mining and network statistical analysis, and set all ncRNAs and their target genes that appeared more than twice in the collected literature as nodes, and finally obtained a ncRNA regulatory network as presented this review (All steps of our approach are represented in Figure 2 and the ncRNA regulatory network thus obtained is shown in Figure 3). Next, based on the number of ncRNA targets and the citations of related ncRNAs, we speculated its potential importance in the gene regulatory network for cancer, determined the final priority. Thereafter, we examined the interaction between the star ncRNAs targets, and the potential biological functions of ncRNAs in CRC. Detailed information on the network composition is shown in Table 1. The filtered nodes, which represent the un-replicated findings, are shown in Table 2. Through this review, we aimed to investigate the role of ncRNA regulatory network in the initiation and progression of CRC. Our review may have implications in future research strategies using ncRNAs in the treatment of CRC and tackling multi-drug resistance.

**TABLE 1:** List of ncRNA-target and the type of interaction present in the network.

ncRNA	Direct target	Type of interaction	PMID	References
CCAT2	miR-145	neg	28964256	Yu et al. (2017a)
CCAT2	BOP1	pos	32805281	Chen et al. (2020c)
ciRS-7	miR-7	pos	32917870	Kristensen et al. (2020)
ciRS-7	miR-7	sponge	28174233	Weng et al. (2017)
CRNDE	EZH2	Pos	28796262	Ding et al. (2017a)
CRNDE	miR-181a-5p	Neg	28086904	Han et al. (2017)
CRNDE	hnRNPUL2	Shuttling neg	28594403	Jiang et al. (2017)
GAS5	YAP		31619268	Ni et al. (2019)
GAS5	miR-222—3p	Sponge	31400607	Liu et al. (2019a)
H19	miR-141	Sponge	30083271	Ren et al. (2018a)
H19	miR-194—5p	Sponge	30451820	Wang et al. (2018a)
HOTAIR	miR-218	Neg	28918035	Li et al. (2017b)
HOTAIR	miR-93	Sponge	32144238	Liu et al. (2020a)
HOTAIR	miR-126	Sponge	31974341	Jiang et al. (2020)
LINC00152	miR-193a-3p	Sponge	27633443	Yue et al. (2016)
LINC00152	miR-139—5p	Sponge	29180678	Bian et al. (2017)
LINC00460	miR-149—5p	Sponge	30092404	Lian et al. (2018)
LINC00460	miR-149—5p	Sponge	33251049	Meng et al. (2020)
LINC00460	miR-150—5p	Sponge	33251049	Meng et al. (2020)
LINC00460	KLF2	Neg	30092404	Lian et al. (2018)
MALAT1	miR-15	Sponge	31097689	Ji et al. (2019)
MALAT1	miR-126—5p	Sponge	30531836	Sun et al. (2019a)
MALAT1	miR-663a	Neg	30154407	Tian et al. (2018)
miR-100	DKK1	Neg	29035371	Lu et al. (2017)
miR-100	DKK3	Neg	29035371	Lu et al. (2017)
miR-100	DKK1	Neg	29094721	Thomas, (2017)
miR-100	DKK3	Neg	29094721	Thomas, (2017)
miR-101	OGT	Neg	30093632	Jiang et al. (2019)
miR-101	EZH2	Neg	30093632	Jiang et al. (2019)
miR-125b	ZNRF3	Neg	29035371	Lu et al. (2017)
miR-125b	RNF43	Neg	29035371	Lu et al. (2017)
miR-125b	APC2	Neg	29035371	Lu et al. (2017)
miR-125b	RNF43	neg	29094721	Thomas, (2017)
miR-125b	APC2	neg	29094721	Thomas, (2017)
miR-1273g-3p	MELK	neg	31358735	Zhao et al. (2019)
miR-1273g-3p	MAGEA3/6	neg	30056111	Wu et al. (2018)
miR-145	MYC	neg	29475734	Zhu et al. (2018a)
miR-145	KLF4	neg	29475734	Zhu et al. (2018a)
miR-145	Nanog	neg	29475734	Zhu et al. (2018a)
miR-149—5p	CUL4A	neg	30092404	Lian et al. (2018)
miR-149—5p	P53	neg	33251049	Meng et al. (2020)
miR-150—5p	P53	neg	33251049	Meng et al. (2020)
miR-150—5p	VEGFA	neg	30250022	Chen et al. (2018)
miR-17—5p	TRIM8	neg	28327152	Mastropasqua et al. (2017)
miR-17—5p	p21	neg	28327152	Mastropasqua et al. (2017)
miR-17—5p	BLNK	neg	30555542	Mai et al. (2018)
miR-181d	PDGFRB	neg	28363996	Jiang and Hermeking, (2017)
miR-181d	FBXL3	neg	28749470	Guo et al. (2017)
miR-181d	PEAK1	neg	29449544	Huang et al. (2018b)
miR-18a	ulk1	neg	28753429	Yu et al. (2017b)
miR-18a	PIAS3	neg	29896300	Ma et al. (2018)
miR-18a	HIF1α	neg	27080303	Ma et al. (2016a)
miR-193a-3p	IL17RD	neg	28600480	Pekow et al. (2017)
miR-193a-3p	ERBB4	neg	27633443	Yue et al. (2016)
miR-193a-3p	IL17RD	neg	28600480	Pekow et al. (2017)
miR-193a-3p	ERBB4	neg	27633443	Yue et al. (2016)
miR-194—5p	MALAT1	neg	31311811	Wu et al. (2019b)
miR-194—5p	SIRT1	neg	30451820	Wang et al. (2018a)
miR-195—5p	YAP	neg	28356122	Sun et al. (2017)
miR-195—5p	NOTCH2	neg	30808369	Lin et al. (2019a)
miR-19a	TGFBR2	neg	27080303	Ma et al. (2016a)
miR-19a	TIA1	neg	28257633	Liu et al. (2017a)
miR-19a	Bim	neg	32591507	Guo et al. (2020a)
miR-19a	TNFAIP3	pos	27991929	Wang et al. (2017a)
miR-200	ZEB1	neg	26455323	Barbáchano et al. (2016)
miR-200b-3p	ZEB1	neg	28837144	Chen et al. (2017a)
miR-200c-3p	ZEB1	neg	28535802	Rigoutsos et al. (2017)
miR-20a	TGFBR2	neg	27080303	Ma et al. (2016a)
miR-20a	VEGFA	neg	27080303	Ma et al. (2016a)
miR-20a	WTX	neg	30631060	Zhu et al. (2019)
miR-21	RASA1	neg	27876571	Yang et al. (2017)
miR-21	IL-6	pos	25994220	Shi et al. (2016)
miR-21	TNF-α	pos	25994220	Shi et al. (2016)
miR-21	IL-17A	pos	25994220	Shi et al. (2016)
miR-21	IL-21	pos	25994220	Shi et al. (2016)
miR-21	PTEN	neg	31918721	Liang et al. (2020a)
miR-21	hMSH2	neg	31918721	Liang et al. (2020a)
miR-214	EZH2	neg	30626446	Xu et al. (2019a)
miR-214	ANLN	neg	30195762	Barbagallo et al. (2018)
miR-214	F23	neg	30195762	Barbagallo et al. (2018)
miR-214	KIF2A	neg	30195762	Barbagallo et al. (2018)
miR-214	IPO7	neg	30195762	Barbagallo et al. (2018)
miR-214	BIRC5	neg	30195762	Barbagallo et al. (2018)
miR-215	NID1	neg	30831320	Rokavec et al. (2019)
miR-215	ZEB2	neg	29187907	Chen et al. (2017b)
miR-215	LGR5	neg	30790680	Ullmann et al. (2019)
miR-221	PTEN	neg	28986522	Antoniali et al. (2017)
miR-221	QKI-5	neg	31416845	Mukohyama et al. (2019)
miR-222	PTEN	neg	28986522	Antoniali et al. (2017)
miR-24	Snora75	neg	28500171	Michael et al. (2017)
miR-24	mt-Nd2	neg	28500171	Michael et al. (2017)
miR-24	VHL	neg	30393198	Jin et al. (2019)
miR-24	PDHB	neg	30393198	Jin et al. (2019)
miR-24	PDHA1	neg	30393198	Jin et al. (2019)
miR-24	DLD	neg	30393198	Jin et al. (2019)
miR-24	IDH3A	neg	30393198	Jin et al. (2019)
miR-25—3p	KLF2	neg	30568162	Zeng et al. (2018a)
miR-25—3p	KLF4	neg	30568162	Zeng et al. (2018a)
miR-25—3p	PTEN	neg	31931030	Wang et al. (2020a)
miR-26a	FUT4	neg	28640257	Li et al. (2017c)
miR-26a	EZH2	neg	30626446	Xu et al. (2019a)
miR-26b	FUT4	neg	28640257	Li et al. (2017c)
miR-26b	EZH2	neg	30626446	Xu et al. (2019a)
miR-27a	ACLY	neg	30393198	Jin et al. (2019)
miR-27a	MDH1B	neg	30393198	Jin et al. (2019)
miR-27a	SDHA	neg	30393198	Jin et al. (2019)
miR-27a	calreticulin	neg	26913599	Colangelo et al. (2016a)
miR-27a	calreticulin	neg	26913609	Colangelo et al. (2016b)
miR-31	Axin1	neg	28870287	Tian et al. (2017)
miR-31	Gsk3b	neg	28870287	Tian et al. (2017)
miR-31	Tgfbr2	neg	28870287	Tian et al. (2017)
miR-31	Bmpr1a	neg	28870287	Tian et al. (2017)
miR-31	Smad4	neg	28870287	Tian et al. (2017)
miR-31	Smad3	neg	28870287	Tian et al. (2017)
miR-31	Dkk1	neg	28870287	Tian et al. (2017)
miR-31	Il7R	neg	30779922	Tian et al. (2019)
miR-31	Il17RA	neg	30779922	Tian et al. (2019)
miR-31	GP130	neg	30779922	Tian et al. (2019)
miR-34a	IL6R	neg	30099074	Öner et al. (2018)
miR-34a	PAI1	neg	30099074	Öner et al. (2018)
miR-34a	PPP1R11	neg	28435028	Li et al. (2017a)
miR-34a	SNHG7	neg	29970122	Li et al. (2018a)
miR-34a	N-MYC	neg	28327152	Mastropasqua et al. (2017)
miR-34a	Pdgfra	neg	28363996	Jiang and Hermeking, (2017)
miR-34a	Axl	neg	28363996	Jiang and Hermeking, (2017)
miR-34a	COL4A2	neg	28363996	Jiang and Hermeking, (2017)
miR-34a	WASF1	neg	28363996	Jiang and Hermeking, (2017)
miR-34a	STC1	neg	28363996	Jiang and Hermeking, (2017)
miR-34a	PDGFRB	neg	28363996	Jiang and Hermeking, (2017)
miR-34a	SIRT1	neg	30312725	Luo et al. (2019)
miR-34a	SIRT1	neg	28943452	Fang et al. (2017)
miR-34a	LMTK3	neg	26739063	Jacob et al. (2016)
miR-34b	Pdgfra	neg	28363996	Jiang and Hermeking, (2017)
miR-34b	Axl	neg	28363996	Jiang and Hermeking, (2017)
miR-34b	COL4A2	neg	28363996	Jiang and Hermeking, (2017)
miR-34b	WASF1	neg	28363996	Jiang and Hermeking, (2017)
miR-34b	STC1	neg	28363996	Jiang and Hermeking, (2017)
miR-34b	PDGFRB	neg	28363996	Jiang and Hermeking, (2017)
miR-34c	LMTK3	neg	26739063	Jacob et al. (2016)
miR-34c	Pdgfra	neg	28363996	Jiang and Hermeking, (2017)
miR-34c	Axl	neg	28363996	Jiang and Hermeking, (2017)
miR-34c	COL4A2	neg	28363996	Jiang and Hermeking, (2017)
miR-34c	WASF1	neg	28363996	Jiang and Hermeking, (2017)
miR-34c	STC1	neg	28363996	Jiang and Hermeking, (2017)
miR-451a	EMSY	neg	28742699	Kelley et al. (2017)
miR-451a	CAB39	neg	28742699	Kelley et al. (2017)
miR-451a	BAP31	neg	30770794	Xu et al. (2019b)
miR-590—5p	YAP	neg	29912317	Yu et al. (2018a)
miR-590—5p	YAP	neg	29429755	Ou et al. (2018)
miR-7	EGFR	neg	28174233	Weng et al. (2017)
miR-7	RAF1	neg	28174233	Weng et al. (2017)
miR-7	FAK	neg	29549306	Zeng et al. (2018b)
miR-7	IGF1R	neg	29549306	Zeng et al. (2018b)
miR-7	EGFR	neg	29549306	Zeng et al. (2018b)
miR-7	YY1	neg	29549306	Zeng et al. (2018b)
NEAT1	ALDH1	pos	33168814	Zhu et al. (2020a)
NEAT1	MYC	pos	33168814	Zhu et al. (2020a)
NEAT1	miR-34a	sponge	30312725	Luo et al. (2019)
NEAT1	DDX5	pos	30185232	Zhang et al. (2018a)
PVT1	MYC	neg	33148262	Shigeyasu et al. (2020)
PVT1	miR-16—5p	neg	32276209	Wu et al. (2020a)
PVT1	Lin28	pos	30076414	He et al. (2019)
PVT1	miR-128	sponge	30076414	He et al. (2019)
SATB2-AS1	SATB2	neg	30858153	Wang et al. (2019a)
SATB2-AS1	SATB2	pos	31492160	Xu et al. (2019c)
UCA1	miR-143	sponge	31955010	Luan et al. (2020)
UCA1	miR-135a	sponge	30195762	Barbagallo et al. (2018)
UCA1	miR-143	sponge	30195762	Barbagallo et al. (2018)
UCA1	miR-214	sponge	30195762	Barbagallo et al. (2018)
UCA1	miR-1271	sponge	30195762	Barbagallo et al. (2018)
ZFAS1	NOP58	pos	32443980	Wu et al. (2020b)
ZFAS1	miR-150—5p	sponge	30250022	Chen et al. (2018)

**TABLE 2:** List of ncRNAs, their targets and the type of interactions, cited by only one scientific article.

ncRNA	Target	Type of interaction	PMID	References
ADAMTS9-AS2	miR-143—3p	sponge	30217729	Xie et al. (2018)
AK000053	miR-508	sponge	29374066	Yan et al. (2018)
AK036396	Ficolin B	neg	32102837	Tian et al. (2020)
ASBEL	ATF3	neg	27791078	Taniue et al. (2016a)
BC032913	TIMP3	pos	28918047	Lin et al. (2017)
BFAL1	miR-155—5p, miR-200a-3p	sponge	31515468	Bao et al. (2019)
BLACAT1	EZH2, p15	Cooperate, neg	28277544	Su et al. (2017)
CALIC	hnRNP-L, AXL	Cooperate, pos	31353791	Kawasaki et al. (2019)
CASC11	hnRNP-K	pos	27012187	Zhang et al. (2016a)
CCAL	AP-2α	neg	25994219	Ma et al. (2016b)
circ101555	miR-597—5p	sponge	31300733	Chen et al. (2019b)
circ5615	miR-149—5p	sponge	32393760	Ma et al. (2020)
circACC1	AMPK	pos	31155494	Li et al. (2019)
circCTNNA1	miR-149—5p	sponge	32699205	Chen et al. (2020b)
circHIPK3	miR-7	neg	29549306	Zeng et al. (2018b)
CYTOR	β-catenin	pos	29606502	Yue et al. (2018)
FARSA-AS1	miR-18b-5p	sponge	33318478	Zhou et al. (2020)
FEZF1-AS1	PKM2	pos	29914894	Bian et al. (2018)
FLANC	pSTAT3	pos	31988194	Pichler et al. (2020)
FOXC2-AS1	FOXC2	pos	32513911	Pan and Xie, (2020)
GLCC1	c-Myc	pos	31375671	Tang et al. (2019a)
GSEC	DHX36	neg	27797375	Matsumura et al. (2017)
HITT	HIF-1α	neg	31784651	Wang et al. (2020b)
HNF1A-AS1	miRNA-34a	sponge	28943452	Fang et al. (2017)
HOXA-AS2	p21, KLF2, EZH2	neg, neg, cooperate	28112720	Ding et al. (2017b)
HOXD-AS1	HOXD3	neg	30823921	Yang et al. (2019)
ITHI4-AS1	JAK1/2, FUS	pos	31557619	Liang et al. (2019a)
KRT7-AS	KRT7	pos	31910722	Chen et al. (2020d)
LDLRAD4-AS1	LDLRAD4	neg	32111819	Mo et al. (2020)
LINC00265	ZMIZ2	pos	31527801	Zhu et al. (2020b)
LINC00659	PI3K	pos	29523145	Tsai et al. (2018)
LINC00858	miR-4766—5p	sponge	31902050	Zhan et al. (2020)
LINC01106	miR-449b-5p	sponge	33067422	Guo et al. (2020b)
LINC01133	SRSF6	sponge	27443606	Kong et al. (2016)
LINC01234	miR-642a-5p	sponge	30755591	Lin et al. (2019b)
LINC01413	hnRNPK, ZEB1	cooperate, pos	31927328	Ji et al. (2020)
LINC01578	NFKBIB	neg	33040438	Liu et al. (2020b)
LINC02023	PTEN	pos	30849479	Wang et al. (2019b)
LINC02418	miR-1273g-3p	sponge	31358735	Zhao et al. (2019)
LINC-UFC1	β-catenin	pos	27195675	Yu et al. (2016)
LINRIS	IGF2BP2	pos	31791342	Wang et al. (2019c)
LNC34a	miR-34a	neg	27077950	Wang et al. (2016a)
LNC-C/EBPβ	Arg1, CYBB, NOS2, ptgs2	neg	30171135	Gao et al. (2018)
LNC-CMPK2	FUBP3	pos	32203166	Gao et al. (2020)
LNC-CRCMSL	HMGB2	shuttling	30575817	Han et al. (2019)
LNC-FAM84B-4	hnRNPK, DUSP1	cooperate, neg	32866608	Peng et al. (2020)
LNC-Gata6	Lgr4, Lgr5	pos	30224759	Zhu et al. (2018b)
LNC-RI	miR-4727—5p	sponge	32279126	Liu et al. (2020c)
LncRNA-APC1	Rab5b	pos	30511962	Wang et al. (2019d)
LNRRIL6	IL-6	pos	31246342	Wang et al. (2019e)
LUCAT1	NCL	binding	33097685	Wu et al. (2020c)
miR-342	FOXM1, FOXQ1	neg	27162244	Weng et al. (2016)
miR-101c	Tet1	neg	28249902	Tie et al. (2017)
miR-105	RAP2C	neg	29238068	Shen et al. (2017a)
miR-106a	WTX	neg	30631060	Zhu et al. (2019)
miR-106b-5p	TRIM8, p21	neg	28327152	Mastropasqua et al. (2017)
miR-10a	ACTG1, MMP14	neg	28383561	Liu et al. (2017b)
miR-124	iASPP	neg	29022915	Liu et al. (2017c)
miR-1249	HMGA2, VEGFA	neg	30755600	Chen et al. (2019c)
miR-125a-3p	FUT5, FUT6	neg	28771224	Liang et al. (2017)
miR-126	SCEL	neg	31974341	Jiang et al. (2020)
miR-126—5p	VEGFA, TWIST, SLUG	neg	30531836	Sun et al. (2019a)
miR-1271	ANLN, BIRC5, IPO7, KIF2A, F23	neg	30195762	Barbagallo et al. (2018)
miR-128	Lin28	neg	30076414	He et al. (2019)
miR-128—3p	Bmi1, MRP5	neg	30890168	Liu et al. (2019b)
miR-130b-3p	PTEN	neg	31931030	Wang et al. (2020a)
miR-135a	ANLN, BIRC5, IPO7, KIF2A, F23	neg	30195762	Barbagallo et al. (2018)
miR-137	GLS1	neg	29730197	Li et al. (2018b)
miR137HG	mir-137	pos	29730197	Li et al. (2018b)
miR-139—5p	PDE4D	neg	27383270	Cao et al. (2016)
miR-141	β-catenin	neg	30083271	Ren et al. (2018a)
miR-141—3p	ZEB1	neg	28535802	Rigoutsos et al. (2017)
miR-143	ANLN, BIRC5, IPO7, KIF2A, F23	neg	30195762	Barbagallo et al. (2018)
miR-143—3p	ITGA6	neg	30217729	Xie et al. (2018)
miR-144	EZH2	neg	30770796	Shi et al. (2019)
miR-146a	c-met	neg	29133238	Bleau et al. (2018)
miR-148a	GP130, IL1R1, IKKα, IKKβ, TNFR2	neg	28960206	Zhu et al. (2017)
miR-149	CDK4/6, XIAP, BCLXL, cyclin D	neg	29061672	Lulla et al. (2017)
miR-149—3p	PDK2	neg	31597953	Liang et al. (2020b)
miR-15	LRP6	neg	31097689	Ji et al. (2019)
miR-150	ZEB1	neg	26455323	Barbáchano et al. (2016)
miR-153	IDO1	neg	29685162	Huang et al. (2018c)
miR-15b	DCLK1	Neg	30449704	Ji et al. (2018)
miR-16—5p	VEGFR1	Pos	32276209	Wu et al. (2020a)
miR17HG	miR-375	Sponge	31409641	Xu et al. (2019d)
miR-181a	SRCIN1	Neg	29739921	Sun et al. (2018)
miR-181a-5p	β-catenin, TCF4	Neg	28086904	Han et al. (2017)
miR-181b	PDCD4	Neg	27647131	Liu et al. (2016)
miR-182	LMTK3	Neg	26739063	Jacob et al. (2016)
miR-187	SOX4, PTK6, NT5E	Neg	26820227	Zhang et al. (2016b)
miR-18b-5p	FARSA	Neg	33318478	Zhou et al. (2020)
miR-193a	Caprin1	Neg	28211508	Teng et al. (2017)
miR-194	VAPA	Neg	29109785	Chang et al. (2017)
miR-195	WEE1, CHK1	Neg	29080751	Kim et al. (2018)
miR-196b-5p	HOXB7, GALNT5	Pos	28533224	Stiegelbauer et al. (2017)
miR-19b	Bim	Neg	32591507	Guo et al. (2020a)
miR-200a-3p	RHEB	Neg	31515468	Bao et al. (2019)
miR-203	BIRC5	Neg	31091026	Okugawa et al. (2019)
miR-205—5p	ZEB1	Neg	29352232	Gulei et al. (2018)
miR-206	Met	Neg	30250188	Xu et al. (2018a)
miR-214—3p	MyD88	Neg	30914411	Shang et al. (2019)
miR-215—5p	EREG, TYMS	Neg	31542354	Chen et al. (2019d)
miR-216b	GALNT1	Neg	29915311	Shan et al. (2018)
miR-218	VOPP1	Neg	28918035	Li et al. (2017b)
miR-22	HuR	Neg	29351796	Liu et al. (2018a)
miR-222—3p	PTEN	Neg	31400607	Liu et al. (2019a)
miR-223	FBX8	Neg	27916606	Wang et al. (2017b)
miR-224	SMAD4	Neg	25804630	Ling et al. (2016)
miR-23a	CS, PDHA1, IDH1, DLD	Neg	30393198	Jin et al. (2019)
miR-23b	LGR5	Neg	28487386	Viswanathan et al. (2017)
miR-301A	BTG1	Neg	28193514	He et al. (2017)
miR-302a	NFIB, CD44	Neg	31754405	Sun et al. (2019b)
miR-30a	ME1	Neg	28475173	Shen et al. (2017b)
miR-30a-5p	LDHA	Neg	28461244	Li et al. (2017d)
miR-320a	PKCγ	Neg	31515469	Aljagthmi et al. (2019)
miR-338—5p	IL-6	Neg	31208913	Xu et al. (2019e)
miR-372/373	SPOP, VDR, SETD7, RELA	Neg	30171794	Wang et al. (2018b)
	TRERF1, ZNF367, MTUS1			
miR-375	RELA, MALT1, NFKBIE	Neg	31409641	Xu et al. (2019d)
	PPP3R1, MAP3K7, CBL			
miR-425—5p	PTEN	Neg	31931030	Wang et al. (2020a)
miR-4260	MCC, SMAD4	Neg	28638476	Xiao et al. (2017)
miR-448	IDO1	Neg	31391111	Lou et al. (2019)
miR-449b-5p	Gli4	Neg	25961913	Wang et al. (2016b)
miR-4727—5p	LIG4	Neg	32279126	Liu et al. (2020c)
miR-4766—5p	PAK2	Neg	31902050	Zhan et al. (2020)
miR-4775	Smad7	Neg	28095858	Zhao et al. (2017)
miR-4802	ATG7	Neg	28753429	Yu et al. (2017b)
miR-486—5p	PLAGL2	Neg	30305607	Liu et al. (2018b)
miR-487b-3p	GRM3	Neg	28114282	Yi et al. (2017)
miR-490—3p	FRAT1	Neg	27037061	Zheng et al. (2016)
miR-494	APC	Neg	29304823	Zhang et al. (2018b)
miR-500a-5p	HDAC2	Neg	30737378	Tang et al. (2019b)
miR-508	SALL4	Neg	29374066	Yan et al. (2018)
miR-514b-3p	FZD4, NTN1	Neg	29880874	Ren et al. (2018b)
miR-514b-5p	CDH1, CLDN1	Neg	29880874	Ren et al. (2018b)
miR-532—3p	ETS1	Neg	31570702	Gu et al. (2019a)
miR-532—5p	TGFBR1	Neg	29971498	Gu et al. (2019b)
miR-550a-3-5p	YAP	Neg	29844307	Choe et al. (2018)
miR-550a-5p	RNF43	neg	25961913	Wang et al. (2016b)
miR-5582—5p	GAB1, CDK2, SHC1	neg	27475256	An et al. (2016)
miR574—5p	APAF1	neg	32784109	Wu et al. (2020d)
miR-597—5p	CDK6, RPA3	neg	31300733	Chen et al. (2019b)
miR-625—3p	MAP2K6	neg	27526785	Rasmussen et al. (2016)
miR-642a-5p	SHMT2	neg	30755591	Lin et al. (2019b)
miR-655—3p	TGFBR2, ICK	neg	28457664	Oshima et al. (2017)
miR-663a	TGFB1, PIK3CD, P53, JUND, P21	neg	30154407	Tian et al. (2018)
miR-675—5p	TP53	neg	31734182	Cen et al. (2020)
miR-6883—5p	CDK4/6, XIAP, BCLXL, cyclin D	neg	29061672	Lulla et al. (2017)
miR-92a-3p	FBXW7, MOAP1	neg	31064356	Hu et al. (2019)
miR-93	ATG12	neg	32144238	Liu et al. (2020a)
miR-944	COP1, MDM2	neg	30393117	Kim et al. (2019b)
MYU	miR-16	neg	27568568	Kawasaki et al. (2016)
N-BLR	miR-141—3p, miR-200c-3p	neg	28535802	Rigoutsos et al. (2017)
OLA1P2	STAT3	neg	26898989	Guo et al. (2016)
Olfr29-ps1	miR-214—3p	sponge	30914411	Shang et al. (2019)
OVAAL	PTBP1	neg	30478051	Sang et al. (2018)
PiHL	RPL11, GRWD1	pos	31903119	Deng et al. (2020)
PINCR	Matrin3	neg	28580901	Chaudhary et al. (2017)
piR-1245	ATF3, BTG1, DUSP1, NFKBIA,FAS, UPP1, SESN2, TP53INP1,MDX1	neg	29382334	Weng et al. (2018)
pirl -54265	p-STAT3, BCL-XL,cleaved-CASP3/7/9	Pos, pos, neg	30555542	Mai et al. (2018)
RAMS11	CBX4	pos	32358485	Silva-Fisher et al. (2020)
RBM5-AS1	CMYC, CCND1, YAP1, SGK1	pos	27520449	Di Cecilia et al. (2016)
RPPH1	TUBB3	pos	31685807	Liang et al. (2019b)
SNHG1	miR-154—5p	sponge	30266084	Xu et al. (2018b)
SNHG5	SPATS2	pos	28004750	Damas et al. (2016)
SNHG6	miR-26a, miR-26b, miR-214	sponge	30626446	Xu et al. (2019a)
SNHG7	miR-216b	sponge	29915311	Shan et al. (2018)
SNHG11	HIF-1α	sponge	33060856	Xu et al. (2020a)
SNHG14	miR-186—5p, EZH2	sponge, pos	31273190	Di et al. (2019)
SNHG15	Slug	pos	29604394	Jiang et al. (2018)
SNORA42	SMAD2	pos	32127004	Xu et al. (2020b)
SNORD12C/78	EIF4A3, LAMC2	pos	32443980	Wu et al. (2020b)
tcon_00012883	MMP1	pos	33135346	Yang et al. (2020b)
TUG1	TWIST1	pos	1988275	Mosthaf et al. (1991)
u50535	CCL20	pos	29970882	Yu et al. (2018b)
UICLM	miR-215	sponge	29187907	Chen et al. (2017b)
UPAT	UHRF1	pos	26768845	Taniue et al. (2016b)
WiNTRLINC1	ASCL2	pos	27292638	Giakountis et al. (2016)
ZNFX1-AS1	miR-144	sponge	30770796	Shi et al. (2019)
δNp63α	miR-320a	pos	31515469	Aljagthmi et al. (2019)

### Classification of ncRNA Networks in Colorectal Cancer

**FIGURE 4:**
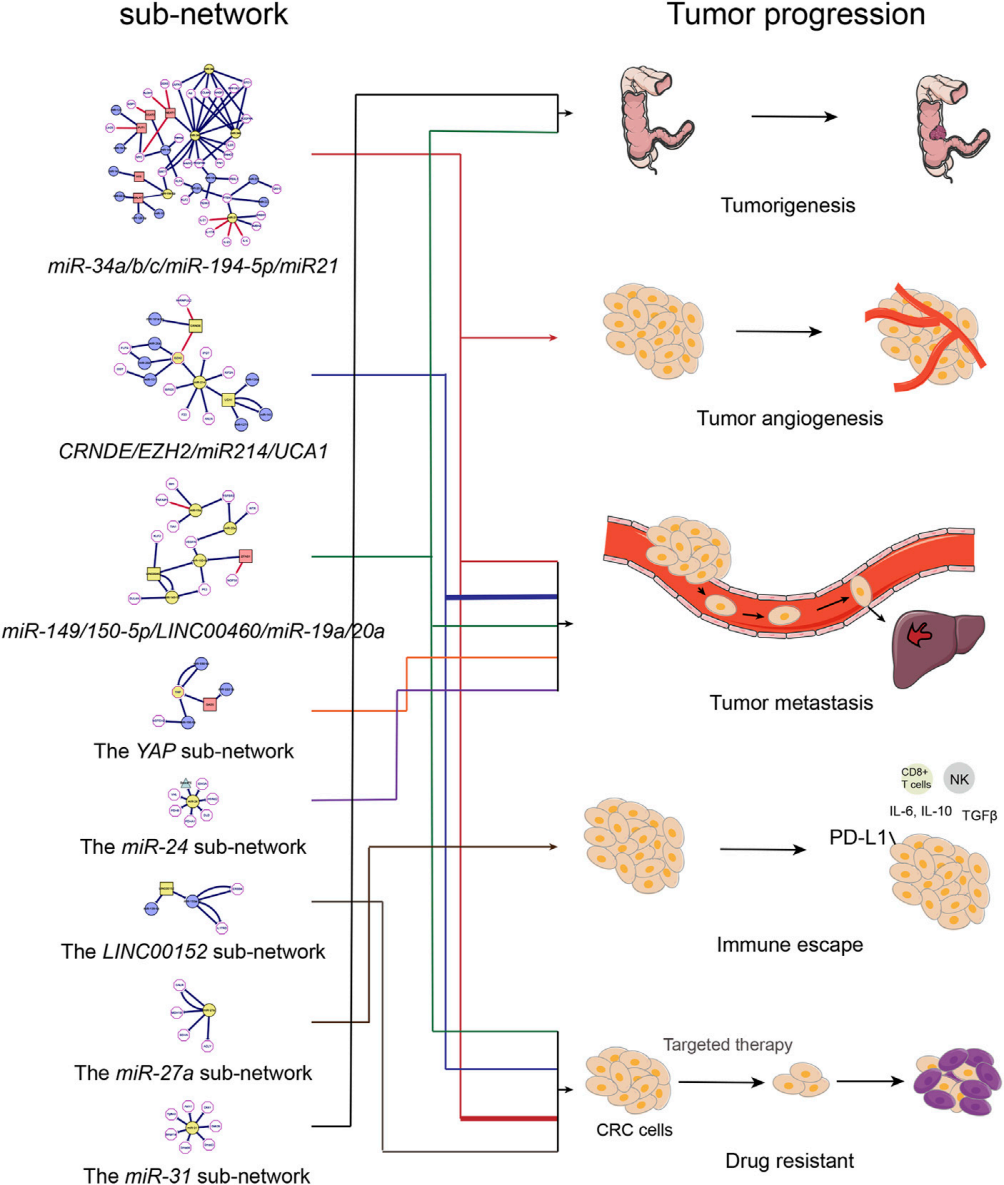
The relationship between ncRNAs network and the progression of colorectal cancer. Diagram of the relationship between ncRNAs sub-network and colorectal cancer progression.

Through the ncRNA network of colorectal cancer, we can clearly observe that the entire network graph is mainly divided into three large sub-networks (Including the miR-34a/b/c/miR-194-5p/miR21 sub-network, the CRNDE/EZH2/miR214/UCA1 sub-network and the miR-149/150-5p/LINC00460/miR-19a/20a sub-network) and a series of small networks (Including small networks with LNC00152, YAP, miR-27a, miR-24, miR-31, miR-7 as the core genes). Among these sub-networks, the miR-34a/b/c/miR-194-5p/miR21 sub-network and the LINC00152 network are mainly related to colorectal cancer chemotherapy resistance, which we call colorectal cancer chemotherapy resistance network; the CRNDE/EZH2/miR214/UCA1 sub-network, the YAP network and miR-24 network are mainly related to the metastasis of colorectal cancer, which we call the colorectal cancer metastasis network. These networks act synergistically and promote the progression of CRC (Figure 4; Table 3).

**TABLE 3:** The relationship between ncRNAs network and the progression of colorectal cancer.

ncRNA network		Tumorigenesis	Tumor angiogenesis	Tumor metastasis	Immune escape	Drug resistant
The miR-34a/b/c/miR-194—5p/miR-21 sub-network	miR-34					PMID 24009080
	miR-194—5p					PMID 30451820
	miR-21					PMID 31918721
The CRNDE/EZH2/UCA1 sub-network	CRNDE					PMID 28086904
	EZH2			PMID 27638307		
	UCA1			PMID 31955010		
The miR-149/150—5p/LINC00460/miR-19a/20a sub-network	miR-149—5p		PMID 30531836			PMID 33251049
	miR-150—5p					PMID 33251049
	LINC00460					PMID 33251049
	miR-19a	PMID 27991929				
	miR-20a			PMID 30631060		
The YAP sub-network	YAP			PMID 28356122		
The miR-24 sub-network	miR-24			PMID 28500171		
The LINC00152 sub-network	LINC00152					PMID 27633443
The miR-27a sub-network	miR-27a				PMID 26913599	
The miR-31 sub-network	miR-31	PMID 28870287				

### Chemotherapy Resistance Network of Colorectal Cancer

Chemotherapy resistance is one of the predominant reasons for the recurrence as well as poor prognosis of colorectal cancer (CRC) patients; ncRNAs reduce chemotherapy resistance of tumors by regulating signaling pathways in the initiation and progression of CRC. We integrated a variety of ncRNAs in CRC chemotherapy resistance and speculated that the combination of ncRNA-targeted inhibitors and chemotherapeutic drugs could be potential agents for improving the therapeutic effect of CRC.

The miR-34a/b/c/miR-194-5p/miR21 sub-network is the core chemotherapeutic resistance network in CRC treatment. The miR-34 family played a critical role in this sub-network by connecting multiple target proteins and lncRNAs. Furthermore, a number of reports show a reduced p53-induced miR-34 expression in CRC cells, and miR-34 can inhibit the occurrence and development of intestinal tumors. Moreover, miR-34 loss is related to tumor progression and chemotherapeutic resistance (Siemens et al., 2013). The mRNA induction in miR-34a/b/c-deficient tumors was enriched in miR-34a/b/c seed-matching sites and mRNAs encoding proteins for Wnt signaling in epithelial-mesenchymal transition (EMT) and stemness such as INHBB, AXL, FGFR1 and PDFGRB, etc. This leads to a decrease in immune cell infiltration and down-regulation of barrier proteins, which in turn promote proliferation and inhibit apoptosis (Jiang and Hermeking, 2017). Meanwhile, studies show that miR-34 mimics can be utilized to stimulate target multiple key pathways, thereby preventing the emergence of drug resistance caused by mutations in a single pathway. The deletion of miR-34a also enhances the effects of TP53 deletion in the early or late stages during CRC initiation and progression. Additionally, miR-34a and TP53 can synergistically inhibit tumor initiation, invasion and metastasis in mouse models of CRC by increasing the levels of target proteins IL6R and PAI1 (Öner et al., 2018). PPP1R11 is also a target of miR-34a, and its product inhibits PP1. In p53-deficient CRC cells, PPP1R11 can activate the phosphorylation of STAT3, and simultaneously, high expression of PPP1R11 can induce EMT, invasion, migration and resistance to 5-fluorouracil under hypoxic conditions. Moreover, miR-34a can reduce the activation of STAT3 in p53-deficient CRC cells by decreasing the expression of PPP1R11, and ultimately inhibit EMT and metastasis of CRC cells (Li et al., 2017a). Thus, we speculate that inhibiting the expression of TP53 and miR-34a in CRC or using miR-34a/b/c replacement therapy may be a potential approach for CRC treatment. The antibodies or small molecule inhibitors to repress miR-34a targeting IL6R and PAI1 are potent promising treatment of CRC in the future.

Additionally, in this sub-network, we can find that KLF4 is significant related to miR-25-3p, and miR-25-3p, as an inhibitor of KLF4, has the effect of promoting the metastasis of CRC (Zeng et al., 2018a). A recent study further showed that MeCP2 bound to METTL14 and enhanced the m^6^A level of KLF4, while m^6^A-modified KLF4 was supposed to be stabilized by IGF2BP2 to increase the expression of KLF4, thereby inhibiting the metastasis of CRC (Wang et al., 2021). Therefore, the development of drugs that simultaneously target to promote the expression of IGF2BP2 and target to inhibit the expression of miR-25-3p may be also an important approach for the treatment of CRC.

SNHG7 (small nucleolar RNA host gene 7), miR-34a and GALNT7 also play an important role in the progression of CRC through the PI3K/AKT/mTOR pathway. SNHG7 can be used as a competitive endogenous RNA (ceRNAs). Along with the sponge miR-34a, it can regulate the level of GALNT7 in CRC and activate the PI3K/AKT/mTOR pathway to promote proliferation and metastasis (Li et al., 2018a). Mastropasqua et al. report that TRIM8 (tripartite motif containing 8) and its regulatory factors including miR-17-5p and miR-106b-5 participate in a feedback loop that controls cell proliferation in CRC by mutual regulation of p53, miR-34a, and N-Myc. In CRC, TRIM8 is a key target that triggers the sensitivity of CRC cells to chemotherapy. TRIM8 restores the function of the p53 tumor suppressor by inactivating the activity of oncoprotein N-Myc in chemotherapy-resistant tumors. Additionally, the silencing of miR-17-5p and miR-106b-5p restore the levels of TRIM8, and effectively promote the tumor suppressor activity of p53 and the transcription of miR-34a, thereby reducing the carcinogenic potential of miR-34a’s target N-Myc. It restores the sensitivity of cells to chemotherapy (Mastropasqua et al., 2017). In addition, LMTK3 (lemur tyrosine kinase 3), an important node in the network diagram, plays an important role in the progression of a variety of cancers (breast cancer, lung cancer, CRC, etc.) (Xu et al., 2014; Xu et al., 2015). In CRC, nuclear LMTK3 interacts with DDX5 to target and regulate the expression of a group of miRNAs (miR-34a, miR-196a2, and miR-182). The tumor suppressor-like miRNAs, miR-34a and miR-182 directly bind to the 3′UTR of LMTK3 mRNA and inhibit its stability and translation, thereby inhibiting the proliferation, invasion, and migration in CRC (Jacob et al., 2016).

In addition to targeting some encoded proteins, miR-34a could also target long-chain non-coding RNAs, and played an important regulatory role in the progression of CRC. NEAT1 (nuclear paraspeckle assembly transcript 1), a long-chain non-coding RNA, is a known oncogene in CRC. For example, NEAT1 can directly interact with its target DDX5 and stabilizes its protein expression. DDX5, thus, activates the Wnt/β-catenin signaling pathway and promotes the progression of CRC (Zhang et al., 2018a). Additionally, some studies show that NEAT1 is associated with 5-FU resistance in CRC. NEAT1 increases H3K27ac enrichment at ALDH1 and c-Myc promoters by altering chromatin remodeling, thereby up-regulating their expression, enhancing the stemness of CRC cells, and promoting 5-FU resistance (Zhu et al., 2020a). Thus, NEAT1 plays an important role in tumor resistance and tumorigenesis in CRC.

However, the effect of NEAT1 on the Wnt/β-catenin signaling pathway is not completely dependent on DDX5, and NEAT1 can also exert carcinogenic effects through miR-34a (Luo et al., 2019). In CRC, NEAT1 acts as a ceRNA that targets miR-34a and regulates its expression, thereby inhibiting the miR-34a/SIRT1 axis. It activates the Wnt/β-catenin signaling pathway, and inhibits miR-34a/SIRT1 feedback loop, which in turn promotes CRC progression, invasion, and metastasis, etc. The above studies show that NEAT1 can be used as a diagnostic marker and is a potential therapeutic target for CRC. Moreover, traditional chemotherapy combined with drugs targeting tumor stem cells provides a new strategy for the treatment of CRC patients CRC patients with high NEAT1 expression. More importantly, the combined network analysis showed that miR-34 may simultaneously target different genes and multiple core pathways in CRC, inhibit EMT, invasion, migration, and proliferation of cancer cells, and prevent the emergence of drug resistance caused by mutations in a single pathway. Therefore, miR-34 replacement therapy could also be a potential option for the treatment of CRC. In addition, targeting a certain pathway regulated by miR-34 for specific effects could also be a potential direction for further research in the treatment of CRC.

In addition to NEAT1, the network diagram also connects a series of other long non-coding RNAs through different target genes. Indeed, there are several studies confirming the role of these long non-coding RNAs in CRC. Therefore, the interaction of different lncRNAs in CRC and their target proteins in this network require in-depth analysis. For example, some studies show that H19 may be the main marker for predicting 5-FU chemotherapy resistance. H19 acts as a ceRNA to target miR-194-5p, and in turn regulates the SIRT1-dependent autophagy pathway which promotes 5-FU chemotherapy resistance in CRC (Wang et al., 2018a). Autophagy is triggered by the target protein of miR-34a, SIRT1 in this network diagram too. Some studies show that H19 acts as a ceRNA sponge of miR-141, activates the β-catenin pathway and promotes stemness as well as chemotherapy resistance of CRC by transferring H19 from exosomes (Ren et al., 2018a). The above studies suggest that conventional chemotherapy combined with targeted therapy can be a potential treatment for 5-FU resistant patients with elevated expression of H19. From the network diagram, we observe that the sub-network highlights another branch of miR-194-5p, which can directly target the lncRNAs-MALAT1 harboring the rs664589 G allele in the nucleus of CRC cells, thereby regulating the nuclear expression of MALAT1 and exerting a tumor suppressor effect (Ren et al., 2018a). Researchers indicate that in CRC, the rs664589 polymorphism of MALAT1 inhibits its affinity to miR-194-5p, resulting in its increased expression, and thus, promotes the development of CRC. Moreover, MALAT1 is primarily induced by YAP1 in CRC and YAP1 interacts with TCF4 and *β*-catenin to regulate the expression of MALAT1 (Sun et al., 2019a). MALAT1 also primarily functions as a competitive endogenous lncRNA in CRC, which targets and regulates the sponging of miR-126-5p, miR-663a, miR-15, and other microRNAs to exhibit a tumor suppressor effect. MALAT1 promotes the expression of VEGFA, SLUG, TWIST, and other metastasis-related molecules by regulating the sponge miR-126-5p; it regulates the angiogenesis and EMT of CRC cells and promotes metastasis (Sun et al., 2019a). Additionally, MALAT1 protects the targets of miR-663a from degradation. MiR-663a and MALAT1 may form a negative feedback loop and affect the progression of CRC (Tian et al., 2018). MALAT1 functions as a ceRNA to regulate the miR-15 family. MiR-15 family inhibits the expression of LRP6 and the activation of the downstream β-catenin signaling pathway. MALAT1 regulates the transcription of the proto-oncogene RUNX2 through the miR-15s/LRP6/β-catenin signaling pathway and thus, regulates the progression of CRC (Ji et al., 2019).

Thereafter, we focus on the last lncRNA-PVT1 of the network diagram. PVT1, a previously unknown transcriptional regulator in CRC, shows a significantly high enhancer activity controlled by epigenetic regulation due to abnormal methylation involved in the occurrence and development of CRC. Enhanced expression of PVT1 is associated with the poor survival in CRC patients with clinical stage II or III status. It also exerts its function as a novel epigenetic enhancer of MYC and responsible for regulating the expression of oncogenic MYC gene (Shigeyasu et al., 2020). Furthermore, PVT1 also functions as a ceRNA to regulate the expression of target genes in the cytoplasm. For example, it can promote the proliferation and invasion of CRC cells by stabilizing Lin28 and interacting with miR-128 (He et al., 2019). Another study reported that PVT1 also promoted the specific binding of RNA-binding proteins (Lin28 and Lin28B) to let-7 by the up-regulation of Lin28 for driving carcinogenic activity of CRC; PVT1 stabilizes and post-transcriptionally regulates Lin28, which targets the Lin28/let-7 axis and promotes tumorigenesis. It is also speculated that the low expression of PVT1 in CRC inhibits EMT and angiogenesis. PVT1 promotes the occurrence of CRC by stabilizing miR-16-5p targeting the VEGFA/VEGFR1/AKT axis. Vascular endothelial growth factor A (VEGFA) is the direct downstream target of miR-16-5p. In the absence of PVT1-miR-16-5p/VEGFA/VEGFR1/AKT, signaling pathway is inactive, thereby inhibiting the progression of CRC (Wu et al., 2020a). In sum, targeting PVT1 may be a potential treatment option for CRC patients.

MiR-21 is also observed as an important multi-target miRNA in this network. Fusobacterium activates the toll-like receptor 4 signaling pathway, which leads to the activation of nuclear factor kappa B (NFκb) and up-regulation of miR-21 expression. Up-regulation of miR-21 reduces the level RAS GTPase (RASA1) and promotes the occurrence and development of CRC (Yang et al., 2017). In mice, silencing miR-21 results in a significant decrease in the expression of pro-inflammatory and cancer-promoting factors (IL6, IL-23, IL-17a and IL-21) and inhibition of tumor proliferation. Studies show that the absence of miR-21 leads to the decrease in Ki67 expression and the inhibition of tumor growth in colitis-associated colon cancer (CAC) mouse, an up-regulation of E-cadherin, and the downregulation of *β*-catenin and SOX9. The deletion of miR-21 increases the expression of its target gene PDCD4, which in turn regulates the activation of NFκb. The deletion of miR-21 also inhibits the activation of STAT3 and Bcl-2 in CAC mice, leading to an increase in tumor cell apoptosis. These studies indicate the regulatory role of miR-21 in the development of CAC caused by colitis (Shi et al., 2016). Moreover, other studies show that miR-21 is correlated with chemotherapeutic resistance of CRC. MiR-21 regulates the expression of downstream targets PTEN and hMSH2, induces tumor cell cycle arrest, inhibits tumor cell proliferation, promotes cell apoptosis, and inhibits migration. MiR-21 targeted therapy can significantly enhance the cytotoxicity of 5-FU in resistant CRC cells and reverse the resistance in CRC just like the exosomal delivery of 5-FU (Liang et al., 2020a).

In the entire chemotherapy resistance network, LINC00152 acting as a ceRNA targets and regulates the expression of miR-193a-3p, antagonizes chemotherapy sensitivity, regulates erb-b2 receptor tyrosine kinase 4 (ERBB4), reduces the phosphorylation of AKT, and thereby reduces resistance to L-OHP (Yue et al., 2016). Similarly, it regulates the expression of NOTCH1 by inhibiting the activity of miR-139-5p, and increasing the resistance of CRC cells to 5-FU (Bian et al., 2017). These studies suggest that the Linc00152/miR-193a-3p/ERBB4/AKT and the LINC00152/miR-139-5p/NOTCH1 signaling axes may provide new insights into CRC resistance mechanisms. In addition, LINC00152 may also be a key tumor suppressor of ulcerative colitis-related CAC. Studies show that miR-193a-3p regulates the expression of IL17RD and controls the downstream EGFR signaling and inhibits the growth of colon cancer (Pekow et al., 2017). Thus, LINC00152 might be a novel potential target in the inflammation-driven CRC patients.

### Metastasis Sub-network of Colorectal Cancer

The metastasis of CRC is the main reason for the poor clinical outcomes and high mortality for CRC patients. The CRNDE/EZH2/UCA1 network is the main component of the metastasis network in CRC. The common target of multiple ncRNAs in this network was the oncogene EZH2 (enhancer of zeste2 polycomb repressive complex 2 subunits). The histone lysine N-methyltransferase encoded by the EZH2 gene is an important part of the PRC2/EED-EZH2 complex, which can methylate the “Lys-9” (H3K9me) and “Lys-27” (H3K27me) of histone H3 and inhibits the transcription of its downstream target genes (McCabe et al., 2012; Hübner et al., 2019). Mutation or over-expression of EZH2 is associated with many types of cancers (breast cancer, prostate cancer, melanoma, bladder cancer, etc.) (Bracken et al., 2003). Presently, many EZH2 targets have been identified. For example, the INK4B-ARF-INK4A tumor suppressor gene locus is a well-known target of EZH2 and its inhibition affects cancer growth and embryonic development (Kheradmand Kia et al., 2009); E-cadherin gene is another critical target of EZH2, and its down-regulation is essential for EMT and metastasis (Luo et al., 2016). Mu Xu et al. report that lncRNA SNHG6 acts as a molecular sponge of miR-26a/b and miR-214, and releases EZH2 by isolating the endogenous microRNA of CRC cells, which mainly regulates the expression of EZH2 in CRC (Xu et al., 2019a). Moreover, EZH2 and its targets H3K27me3, P14ARF, P15INK4b, P16INK4a and E-cadherin are involved in the carcinogenic effect of SNHG6 in CRC and regulate the EMT (Xu et al., 2019a).

In the sub-network of EZH2, O-glycosylation, is a unique post-translational modification (PTM), which participates in CRC metabolic reprogramming. The level of O-glycosylation increases in metastatic CRC tissues or cells. The expression of miR-101 reduces, while the expression of o-GlcNAc acyltransferase (OGT) and EZH2, which are regulated by miR-101, increases significantly. The down-regulation of miR-101 promotes O-GlcNAcylation, and the increased O-GlcNAcylation further enhances the stability and function of the EZH2 protein. O-GlcNAcylation and EZH2-mediated H3K27me3 modification of the miR-101 promoter region further reduce the expression of miR-101, consequently, miR-101/O-GlcNAcylation/EZH2 signals form a feedback loop that inhibits metastasis and eventually inhibits the invasion of CRC cells and regulate the EMT (Jiang et al., 2019). Thus, EZH2 has extremely high potential as a new target for CRC treatment.

Another important network node in the network diagram was CRNDE. CRNDE is located on human chromosome 16 and is highly expressed in a variety of cancers including CRC. CRNDE binds to EZH2, which in turn, can directly bind to DUSP5 and CDKN1A promoter regions, and induce histone H3 lysine 27 trimethylation (H3K27me3) modification in DLD1 and HCT116 cells (Ding et al., 2017a). This also inhibits dual specific phosphatase 5 (DUSP5) and CDKN1A expression and promotes the development of CRC (Ding et al., 2017a). In addition, CRNDE is also related to microRNAs. Han et al. found that miR-181a-5p could be used as the inhibitory target of CRNDE. *β*-catenin and TCF4 are inhibitory targets of miR-181a-5p and repress the Wnt/*β*-catenin signaling pathway. In CRC cell lines, CRNDE promotes CRC cell proliferation and chemotherapy resistance through the Wnt/*β*-catenin signaling pathway mediated by miR-181a-5p (Han et al., 2017). Thus, it warrants further studies to investigate the regulatory mechanism of CRNDE as a potential target in the therapy strategy and resistance of CRC.

Another ncRNA in the network diagram was the lncRNAs UCA1 (urothelial cancer associated 1). The presence of UCA1 in exosomes is verified, but its role and clinical applicability in CRC remain unclear. Barbagallo reported that UCA1 is upregulated in CRC biopsy (Barbagallo et al., 2018). In serum exosomes, the expression of UCA1 is regulated by an activating MAPK signal. UCA1 isolates miR-135a, miR-143, miR-214, and miR-1271 to protect ANLN, BIRC5, IPO7, KIF2A, KIF23 and other actin and cytoskeleton related proteins from miRNA-induced degradation, and thus, regulates their expression and promotes the progression of CRC and other key biological processes (Barbagallo et al., 2018). Luan et al. also demonstrate that UCA1 is upregulated in the serum exosomes of patients with CRC. UCA1 is packaged into exosomes which are transferred to CRC cells. As a ceRNA, UCA1 regulates the expression of MYO6 through miR-143, enhances cell proliferation and migration, and exerts essential functions in the tumor progression of CRC (Luan et al., 2020). Taken together, these reports suggest that UCA1 may be a potential new clinical biomarker for CRC.

From Figure 4, we observe that in addition to the CRNDE/EZH2/miR214/UCA1 network, the YAP sub-network and the miR-24 sub-network also play indispensable roles during the metastasis of CRC. YAP1 is upregulated through a variety of biological mechanisms and has a carcinogenic effect in a variety of tumors. As the core sub-network of CRC metastasis, YAP connected multiple ncRNAs such as GAS5, miR375, and circ1662, etc. The inactivation of YAP1 is required in cell-cell contact inhibition and act as a transcriptional co-activator to mediate the biological functions of the Hippo pathway (Zhao et al., 2007). It should be noted that lncRNA GAS5 (growth arrest-specific 5), a tumor suppressor in CRC as a ceRNA of miR-222-3p, regulates the expression of Beclin1, LC3B, and PTEN by targeting miR-222-3p/GAS5 phosphatase and PTEN signaling pathways, thereby inhibiting CRC cell migration and invasion, and promotes autophagy (Liu et al., 2019a). Notably, GAS5 as well as its target YAP are intently linked to m^6^A modification. GAS5 directly interacts with the WW domain of YAP to promote the transfer of endogenous YAP from the nucleus to the cytoplasm, as well as its phosphorylation and its subsequent ubiquitin-mediated degradation which leads to tumor suppression (Ni et al., 2019). Interestingly, YTHDF3 selectively bound to GAS5 which was modified by m^6^A and promoted the degradation of GAS5 in an m^6^A-dependent manner. Meanwhile, GAS5 negatively regulated the expression of YAP, and YAP could bind to the promoter region of YTHDF3 to promote the transcription of YTHDF3, in other words, YTHDF3-GAS5-YAP-YTHDF3 formed a positive feedback loop and promoted the metastasis of CRC in an m^6^A-dependent manner (Ni et al., 2019). Moreover, YAP not only regulated the expression of YTHDF3, m^6^A modified YAP also directly bound to IGF2BP2, and stabilized YAP promoted the occurrence of CRC by up-regulating the expression of ErbB2 (Cui et al., 2021). In addition to directly regulating the expression of YAP mRNA, m^6^A modification is supposed to indirectly regulate the expression of YAP1 protein. Chen et al. illustrated that the overexpression of METTL14 increased the m^6^A level of primiR-375, and the m^6^A-modified primiR-375 was transformed into premiR-375 under the action of DGCR8, thereby promoting the expression of miR-375 in CRC. Thereafter, elevated miR-375 suppressed the expression of YAP1, and ultimately inhibited the metastasis of CRC (Chen et al., 2020a). Interestingly, the expression of YAP1 protein is not only regulated by m^6^A-modified miRNA, but also by m^6^A-modified circRNA. Studies have shown that METTL3 induced the expression of circ1662 by installing m^6^A modification in the circ1662 flanking reverse complement sequence. The overexpression of circ1662 promoted the transport of YAP1 protein to the nucleus and reduced the level of YAP1 protein in the cytoplasm and ultimately accelerated the metastasis of CRC (59).

MiR-590-5p inhibits the YAP expression by directly targeting its 3′UTR, thereby inhibiting intestinal inflammation and tumorigenesis of CRC cells (60). Ou et al., also validated the existence of the miR-590-5p/YAP axis. MiR-590-5p is a miRNA with density-sensitive property. The high density of CRC cells upregulates the expression of the RNase III endonuclease DICER1, which in turn promotes the biosynthesis of miR-590-5p and ultimately inhibits YAP expression (Ou et al., 2018). This also suggests that the miR-590-5p/YAP axis may be an important specific therapeutic target contributing to the pathogenesis of CRC. Furthermore, miR-590-5p itself may also serve as a therapeutic potential target for CRC patients. miR-590-5p is a hypoxia-sensitive miRNA and inhibits the expression of RECK, which promotes the invasion and metastasis of CRC cells by activating matrix metalloproteinases (MMPs) and filamentous processes in vitro, and consequently promotes tumor cell proliferation (Kim et al., 2019a). Moreover, Nuclear factor 90 (NF90), a direct target of miR-590-5p, is a positive regulator of vascular endothelial growth factor (VEGF) mRNA stability and protein synthesis. The NF90/VEGFA signaling axis can inhibit angiogenesis and metastasis in CRC (Zhou et al., 2016). In contrast, MiR-195, in the YAP sub-network, is an inhibitor of the Hippo-YAP signaling pathway. There are two conserved miR-195-5p homologous sites at the 3′UTR of YAP mRNA. MiR-195-5p inhibits EMT and blocks Hippo signaling, thereby inhibiting the proliferation, migration, invasion and epithelial-mesenchymal transition (EMT) of CRC cells (Sun et al., 2017). In addition, miR-195-5p can also regulate the expression of NOTCH2 in a post-transcriptional manner (Lin et al., 2019a).

Previous publications show that the miRNAs are usually organized in clusters (within 3 kb) in the genome and have the characteristics of a regulatory network that controls tumor metabolism. MiRNA clusters play essential roles in tumor progression by coordinating or inhibiting multiple target genes. The coordinated regulation of miRNA clusters may cause rapid switching of the metabolic signaling networks in CRC cells. Jin et al. report a cluster consisting of miR-23a, miR-27a and miR-24 induced by hypoxia conditions in CRC cells, which promotes glycolysis by regulating the related gene networks. Inhibition of miR-23a, miR-24, and miR-27a under hypoxic conditions weaken the stimulating effect of reduced oxygen on glycolysis-related genes along with the inhibitory effect on tricarboxylic acid cycle-related genes including PDHB, PDHA1, IDH2, DLD, and IDH3A. Moreover, miR-24 promotes the expression of HIF-1α by targeting VHL, forming a double negative feedback loop and exhibits the strongest regulatory effect. Thus, it shows that the miR-23a/27a/24 cluster promotes the progression of CRC through metabolism reprogramming (Jin et al., 2019).

### Other Sub-network in Colorectal Cancer

Among other sub-networks, miR-149/150-5p/LINC00460/miR-19a/20a occupies a major position. It covers tumorigenesis, metastasis and chemotherapy resistance of CRC. In the sub-network, LINC00460, acting as a vital ncRNA, linked multiple miRNAs such as miR-149-5p, miR-150-5p, etc. Studies show that LINC00460 has a carcinogenic effect on CRC. It recruits EZH2 (enhancer of zeste homolog 2, EZH2) and H3K27me3 to the tumor suppressor KLF2 promoter in the nucleus. Thereby, it epigenetically inhibits the expression and inactivation of KLF2 (Lian et al., 2018). LINC00460, as a molecular sponge of miR-149-5p, antagonizes its ability to inhibit the translation of cullin4A (CUL4A) protein and regulates the occurrence of CRC. Thereafter, LINC00460 directly interacted with IGF2BP2 and DHX9 and combined with m^6^A-modified HGMA1 mRNA to enhance the stability of HGMA1 and ultimately promoted the metastasis of CRC (Hou et al., 2021). Notably, LINC00460 may also be a promising therapeutic target involved in chemotherapeutic resistance of CRC. Meng et al. found that LINC00460-miR-149/150-5p-mutant p53 feedback loop is associated with oxaliplatin resistance of CRC. Similarly, LINC00460 promotes oxaliplatin resistance by isolating miR-149-5p/miR-150-5p and upregulating the expression of the target p53 (Meng et al., 2020). In addition to LINC00460, the circCTNNA1 also acts as a ceRNA competitive sponging miR-149-5p to counteract its inhibitory effect on the downstream target FOXM1, thereby promoting the progression of CRC (Chen et al., 2020b). Similarly, circ5615 binds to miR-149-5p, exerting miR-149-5p sponge effect, upregulating TNKS, and subsequently promoting the progression of CRC through the Wnt/β-catenin signaling pathway (Ma et al., 2020). Thus, the carcinogenic functions of LINC00460 or circCTNNA1 as ceRNA in CRC were validated, which suggested that these indicators might be potential and valuable therapeutic targets in CRC treatment and multi-drug resistance.

Besides miR-149-5p, miR-150-5p, miR-19a/20 as critical parts of the sub-network, miR-200 family including miR-200, miR-200b-3p, and miR-200c-3p was found involved in the regulation of ZEB1 and XIST, etc. Interestingly, ZEB1 acted as one of the downstream targets of miR-200b-3p, the combination of XIST and miR-200b-3p disrupts the combination of miR-200b-3p and ZEB1. Meanwhile, XIST can also act as a sponge of miR-200b-3p to promote the expression of ZEB1 and thus promote the progression and metastasis of CRC (71). Importantly, recent report supports that METTL14 can increase the m^6^A level of XIST and decrease the expression of XIST in a YTHDF2-dependent regulation manner. The decrease of XIST expression promotes the expression of miR-200b-3p by directly binding to miR-200b-3p (Yang et al., 2020a). Thus, these findings indicated that linking m^6^A-modified XIST with miR-200 and miR-200c-3p might provide novel directions and approach for excavating the potential targets for CRC therapy.

Notably, another lncRNA, ZFAS1 is highly expressed in CRC tissues and cells. Moreover, as a miR-150-5p sponge, it targets and regulates the expression of its downstream VEGFA, and promotes the progression of CRC by promoting miR150-5p-mediated VEGFA/VEGFR2/Akt/mTOR signaling pathway and EMT (Chen et al., 2018). In addition, studies show that ZFAS1 promotes CRC by small nucleolar RNA-mediated 2′-O methylation through NOP58 recruitment and plays essential roles through the ZFAS1-NOP58-SNORD12C/78-EIF4A3/LAMC2 signaling axis (Wu et al., 2020b). Collectively, these researches broaden our spectrum and lay a solid foundation for further excavating the crosstalk functions between epigenetic modification and ncRNAs during the early prediction and therapy of CRC.

## Conclusions and Perspectives

During the past few decades, extensive promotions have been made to explore the biological functions of ncRNAs in the involvement of tumorigenesis and progression of various types of tumors including CRC. In this review, we analyzed the regulation network and sub-networks related to ncRNAs involved in the progression, metastasis and chemoresistance of CRC via transcriptional and post-transcriptional epigenetic modification levels. Among the networks, the miR-34a/b/c/miR-194-5p/miR21 sub-network showed a direct relationship with oxaliplatin resistance for CRC therapy. Meanwhile, the CRNDE/EZH2/UCA1 sub-network had a significant association with metastasis and progression of CRC. Furthermore, we analyzed the regulatory manner of the core m^6^A regulators with m^6^A-related ncRNAs as exemplified by YTHDF3-GAS5-YAP, IGF2BP2-YAP-ErbB2, METTL14-YTHDF2-XIST, MeCP2/METTL14-KLF4, LINC00460/IGF2BP2/DHX9-HMGA1 signaling axis in CRC progression.

Thus, the crosstalk and regulation network of m^6^A modifications associated modulators and ncRNAs provide a novel direction for exploring the underlying regulatory mechanisms of gene expression in CRC development.

Until now, multiple ncRNAs associated epigenetic m^6^A modification modulators has been found acting as potential biomarkers and targets for CRC therapeutic interventions. However, these indicators have not been effectively developed and applied for the CRC therapy, partly due to exceeding targets for each regulator. For example, IGF2BP1, IGF2BP2 and IGF2BP3 has an enrichment of 3747, 3211, and 3914 high confidence downstream targets, respectively (Huang et al., 2018a). These targets and cellular biological pathways were closely connected to form a huge ncRNAs regulatory network. Thus, targeting multiple dysregulated targets in the m^6^A associated ncRNAs network holds an important potential direction contributing for CRC therapy. Developing highly specific and selective small-molecule inhibitors targeting m^6^A regulators and associated ncRNAs demand urgently for inter-individual precision therapy of CRC. Overall, the regulatory network provides a foundation for further study of ncRNAs, which also provide critical possibilities for clinical treatment through their associations with m^6^A epigenetic modifications that warrants further investigations for CRC.

## References

[B1] Aljagthmi A. A. Hill N. T. Cooke M. Kazanietz M. G. Abba M. C. Long W. (2019). ΔNp63α Suppresses Cells Invasion by Downregulating PKCγ/Rac1 Signaling through miR-320a. Cell Death Dis 10 (9), 680. 10.1038/s41419-019-1921-6 31515469 PMC6742631

[B2] An H.-J. Kwak S.-Y. Yoo J.-O. Kim J.-S. Bae I.-H. Park M.-J. (2016). Novel miR-5582-5p Functions as a Tumor Suppressor by Inducing Apoptosis and Cell Cycle Arrest in Cancer Cells through Direct Targeting of GAB1, SHC1, and CDK2. Biochim. Biophys. Acta (Bba) - Mol. Basis Dis. 1862 (10), 1926–1937. 10.1016/j.bbadis.2016.07.017 27475256

[B3] Antoniali G. Serra F. Lirussi L. Tanaka M. D’Ambrosio C. Zhang S. (2017). Mammalian APE1 Controls miRNA Processing and its Interactome Is Linked to Cancer RNA Metabolism. Nat. Commun. 8 (1), 797. 10.1038/s41467-017-00842-8 28986522 PMC5630600

[B4] Bao Y. Tang J. Qian Y. Sun T. Chen H. Chen Z. (2019). Long Noncoding RNA BFAL1 Mediates Enterotoxigenic Bacteroides Fragilis-Related Carcinogenesis in Colorectal Cancer via the RHEB/mTOR Pathway. Cel Death Dis 10 (9), 675. 10.1038/s41419-019-1925-2 PMC6742644 31515468

[B5] Barbáchano A. Fernández-Barral A. Pereira F. Segura M. F. Ordóñez-Morán P. Carrillo-de Santa Pau E. (2016). SPROUTY-2 Represses the Epithelial Phenotype of colon Carcinoma Cells via Upregulation of ZEB1 Mediated by ETS1 and miR-200/miR-150. Oncogene 35 (23), 2991–3003. 10.1038/onc.2015.366 26455323

[B6] Barbagallo C. Brex D. Caponnetto A. Cirnigliaro M. Scalia M. Magnano A. (2018). LncRNA UCA1, Upregulated in CRC Biopsies and Downregulated in Serum Exosomes, Controls mRNA Expression by RNA-RNA Interactions. Mol. Ther. - Nucleic Acids 12, 229–241. 10.1016/j.omtn.2018.05.009 30195762 PMC6023947

[B7] Bhan A. Mandal S. S. (2015). LncRNA HOTAIR: A Master Regulator of Chromatin Dynamics and Cancer. Biochim. Biophys. Acta (Bba) - Rev. Cancer 1856 (1), 151–164. 10.1016/j.bbcan.2015.07.001 PMC4544839 26208723

[B8] Bian Z. Zhang J. Li M. Feng Y. Wang X. Zhang J. (2018). LncRNA-FEZF1-AS1 Promotes Tumor Proliferation and Metastasis in Colorectal Cancer by Regulating PKM2 Signaling. Clin. Cancer Res. 24 (19), 4808–4819. 10.1158/1078-0432.ccr-17-2967 29914894

[B9] Bian Z. Zhang J. Li M. Feng Y. Yao S. Song M. (2017). Long Non-coding RNA LINC00152 Promotes Cell Proliferation, Metastasis, and Confers 5-FU Resistance in Colorectal Cancer by Inhibiting miR-139-5p. Oncogenesis 6 (11), 395. 10.1038/s41389-017-0008-4 29180678 PMC5868057

[B10] Bleau A.-M. Redrado M. Nistal-Villan E. Villalba M. Exposito F. Redin E. (2018). miR-146a Targets C-Met and Abolishes Colorectal Cancer Liver Metastasis. Cancer Lett. 414, 257–267. 10.1016/j.canlet.2017.11.008 29133238

[B11] Bracken A. P. Pasini D. Capra M. Prosperini E. Colli E. Helin K. (2003). EZH2 Is Downstream of the pRB-E2f Pathway, Essential for Proliferation and Amplified in Cancer. EMBO J. 22 (20), 5323–5335. 10.1093/emboj/cdg542 14532106 PMC213796

[B12] Cao B. Wang K. Liao J. M. Zhou X. Liao P. Zeng S. X. (2016). Inactivation of Oncogenic cAMP-specific Phosphodiesterase 4D by miR-139-5p in Response to P53 Activation. Elife 5. 10.7554/eLife.15978 PMC4959878 27383270

[B13] Cen B. Lang J. D. Du Y. Wei J. Xiong Y. Bradley N. (2020). Prostaglandin E2 Induces miR675-5p to Promote Colorectal Tumor Metastasis via Modulation of P53 Expression. Gastroenterology 158 (4), 971–984. 10.1053/j.gastro.2019.11.013 31734182 PMC7062589

[B14] Chang H.-Y. Ye S.-P. Pan S.-L. Kuo T.-T. Liu B. C. Chen Y.-L. (2017). Overexpression of miR-194 Reverses HMGA2-Driven Signatures in Colorectal Cancer. Theranostics 7 (16), 3889–3900. 10.7150/thno.20041 29109785 PMC5667412

[B15] Chaudhary R. Gryder B. Woods W. S. Subramanian M. Jones M. F. Li X. L. (2017). Prosurvival Long Noncoding RNA PINCR Regulates a Subset of P53 Targets in Human Colorectal Cancer Cells by Binding to Matrin 3. Elife 6. 10.7554/eLife.23244 PMC5470874 28580901

[B16] Chen B. Dragomir M. P. Fabris L. Bayraktar R. Knutsen E. Liu X. (2020). The Long Noncoding RNA CCAT2 Induces Chromosomal Instability through BOP1-AURKB Signaling. Gastroenterology 159 (6), 2146–2162. 10.1053/j.gastro.2020.08.018 32805281 PMC7725986

[B17] Chen C. Yuan W. Zhou Q. Shao B. Guo Y. Wang W. (2021). N6-methyladenosine-induced Circ1662 Promotes Metastasis of Colorectal Cancer by Accelerating YAP1 Nuclear Localization. Theranostics 11 (9), 4298–4315. 10.7150/thno.51342 33754062 PMC7977475

[B18] Chen D.-l. Chen L.-z. Lu Y.-x. Zhang D.-s. Zeng Z.-l. Pan Z.-z. (2017). Long Noncoding RNA XIST Expedites Metastasis and Modulates Epithelial-Mesenchymal Transition in Colorectal Cancer. Cel Death Dis 8 (8), e3011. 10.1038/cddis.2017.421 PMC5596599 28837144

[B19] Chen D.-l. Lu Y.-x. Zhang J.-x. Wei X.-l. Wang F. Zeng Z.-l. (2017). Long Non-coding RNA UICLM Promotes Colorectal Cancer Liver Metastasis by Acting as a ceRNA for microRNA-215 to Regulate ZEB2 Expression. Theranostics 7 (19), 4836–4849. 10.7150/thno.20942 29187907 PMC5706103

[B20] Chen P. Yao Y. Yang N. Gong L. Kong Y. Wu A. (2020). Circular RNA circCTNNA1 Promotes Colorectal Cancer Progression by Sponging miR-149-5p and Regulating FOXM1 Expression. Cel Death Dis 11 (7), 557. 10.1038/s41419-020-02757-7 PMC7376054 32699205

[B21] Chen R.-X. Chen X. Xia L.-P. Zhang J.-X. Pan Z.-Z. Ma X.-D. (2019). N6-methyladenosine Modification of circNSUN2 Facilitates Cytoplasmic export and Stabilizes HMGA2 to Promote Colorectal Liver Metastasis. Nat. Commun. 10 (1), 4695. 10.1038/s41467-019-12651-2 31619685 PMC6795808

[B22] Chen S. Su T. Zhang Y. Lee A. He J. Ge Q. (2020). Fusobacterium Nucleatum Promotes Colorectal Cancer Metastasis by Modulating KRT7-AS/KRT7. Gut Microbes 11 (3), 511–525. 10.1080/19490976.2019.1695494 31910722 PMC7524269

[B23] Chen S. Yue T. Huang Z. Zhu J. Bu D. Wang X. (2019). Inhibition of Hydrogen Sulfide Synthesis Reverses Acquired Resistance to 5-FU through miR-215-5p-EREG/TYMS axis in colon Cancer Cells. Cancer Lett. 466, 49–60. 10.1016/j.canlet.2019.09.006 31542354

[B24] Chen X. Xu M. Xu X. Zeng K. Liu X. Sun L. (2020). METTL14 Suppresses CRC Progression via Regulating N6-methyladenosine-dependent Primary miR-375 Processing. Mol. Ther. 28 (2), 599–612. 10.1016/j.ymthe.2019.11.016 31839484 PMC7001002

[B25] Chen X. Zeng K. Xu M. Hu X. Liu X. Xu T. (2018). SP1-induced lncRNA-ZFAS1 Contributes to Colorectal Cancer Progression via the miR-150-5p/VEGFA axis. Cel Death Dis 9 (10), 982. 10.1038/s41419-018-0962-6 PMC6155123 30250022

[B26] Chen X. Zeng K. Xu M. Liu X. Hu X. Xu T. (2019). P53-induced miR-1249 Inhibits Tumor Growth, Metastasis, and Angiogenesis by Targeting VEGFA and HMGA2. Cel Death Dis 10 (2), 131. 10.1038/s41419-018-1188-3 PMC6372610 30755600

[B27] Chen Z. Ren R. Wan D. Wang Y. Xue X. Jiang M. (2019). Hsa_circ_101555 Functions as a Competing Endogenous RNA of miR-597-5p to Promote Colorectal Cancer Progression. Oncogene 38 (32), 6017–6034. 10.1038/s41388-019-0857-8 31300733

[B28] Choe M. H. Yoon Y. Kim J. Hwang S.-G. Han Y.-H. Kim J.-S. (2018). miR-550a-3-5p Acts as a Tumor Suppressor and Reverses BRAF Inhibitor Resistance through the Direct Targeting of YAP. Cel Death Dis 9 (6), 640. 10.1038/s41419-018-0698-3 PMC5974323 29844307

[B29] Colangelo T. Polcaro G. Ziccardi P. Muccillo L. Galgani M. Pucci B. (2016). The miR-27a-Calreticulin axis Affects Drug-Induced Immunogenic Cell Death in Human Colorectal Cancer Cells. Cel Death Dis 7, e2108. 10.1038/cddis.2016.29 PMC4849155 26913599

[B30] Colangelo T. Polcaro G. Ziccardi P. Pucci B. Muccillo L. Galgani M. (2016). Proteomic Screening Identifies Calreticulin as a miR-27a Direct Target Repressing MHC Class I Cell Surface Exposure in Colorectal Cancer. Cel Death Dis 7, e2120. 10.1038/cddis.2016.28 PMC4849154 26913609

[B31] Cui J. Tian J. Wang W. He T. Li X. Gu C. (2021). IGF2BP2 Promotes the Progression of Colorectal Cancer through a YAP-dependent Mechanism. Cancer Sci. 10.1111/cas.15083 PMC8486198 34309973

[B32] Damas N. D. Marcatti M. Côme C. Christensen L. L. Nielsen M. M. Baumgartner R. (2016). SNHG5 Promotes Colorectal Cancer Cell Survival by Counteracting STAU1-Mediated mRNA Destabilization. Nat. Commun. 7, 13875. 10.1038/ncomms13875 28004750 PMC5192221

[B33] Dang Q. Shao B. Zhou Q. Chen C. Guo Y. Wang G. (2021). RNA N6-Methyladenosine in Cancer Metastasis: Roles, Mechanisms, and Applications. Front. Oncol. 11, 681781. 10.3389/fonc.2021.681781 34211849 PMC8239292

[B34] Deng X. Li S. Kong F. Ruan H. Xu X. Zhang X. (2020). Long Noncoding RNA PiHL Regulates P53 Protein Stability through GRWD1/RPL11/MDM2 axis in Colorectal Cancer. Theranostics 10 (1), 265–280. 10.7150/thno.36045 31903119 PMC6929633

[B35] Di Cecilia S. Zhang F. Sancho A. Li S. Aguiló F. Sun Y. (2016). RBM5-AS1 Is Critical for Self-Renewal of Colon Cancer Stem-like Cells. Cancer Res. 76 (19), 5615–5627. 10.1158/0008-5472.can-15-1824 27520449 PMC5050123

[B36] Di W. Weinan X. Xin L. Zhiwei Y. Xinyue G. Jinxue T. (2019). Long Noncoding RNA SNHG14 Facilitates Colorectal Cancer Metastasis through Targeting EZH2-Regulated EPHA7. Cel Death Dis 10 (7), 514. 10.1038/s41419-019-1707-x PMC6609685 31273190

[B37] Ding J. Li J. Wang H. Tian Y. Xie M. He X. (2017). Long Noncoding RNA CRNDE Promotes Colorectal Cancer Cell Proliferation via Epigenetically Silencing DUSP5/CDKN1A Expression. Cel Death Dis 8 (8), e2997. 10.1038/cddis.2017.328 PMC5596537 28796262

[B38] Ding J. Xie M. Lian Y. Zhu Y. Peng P. Wang J. (2017). Long Noncoding RNA HOXA-AS2 Represses P21 and KLF2 Expression Transcription by Binding with EZH2, LSD1 in Colorectal Cancer. Oncogenesis 6 (1), e288. 10.1038/oncsis.2016.84 28112720 PMC5294247

[B39] Fang C. Qiu S. Sun F. Li W. Wang Z. Yue B. (2017). Long Non-coding RNA HNF1A-AS1 Mediated Repression of miR-34a/SIRT1/p53 Feedback Loop Promotes the Metastatic Progression of colon Cancer by Functioning as a Competing Endogenous RNA. Cancer Lett. 410, 50–62. 10.1016/j.canlet.2017.09.012 28943452

[B40] Gao Q. Zhou R. Meng Y. Duan R. Wu L. Li R. (2020). Long Noncoding RNA CMPK2 Promotes Colorectal Cancer Progression by Activating the FUBP3-C-Myc axis. Oncogene 39 (19), 3926–3938. 10.1038/s41388-020-1266-8 32203166

[B41] Gao Y. Sun W. Shang W. Li Y. Zhang D. Wang T. (2018). Lnc-C/Ebpβ Negatively Regulates the Suppressive Function of Myeloid-Derived Suppressor Cells. Cancer Immunol. Res. 6 (11), 1352–1363. 10.1158/2326-6066.cir-18-0108 30171135

[B42] Giakountis A. Moulos P. Zarkou V. Oikonomou C. Harokopos V. Hatzigeorgiou A. G. (2016). A Positive Regulatory Loop between a Wnt-Regulated Non-coding RNA and ASCL2 Controls Intestinal Stem Cell Fate. Cel Rep. 15 (12), 2588–2596. 10.1016/j.celrep.2016.05.038 27292638

[B43] Gu C. Cai J. Xu Z. Zhou S. Ye L. Yan Q. (2019). MiR-532-3p Suppresses Colorectal Cancer Progression by Disrupting the ETS1/TGM2 axis-mediated Wnt/β-Catenin Signaling. Cel Death Dis 10 (10), 739. 10.1038/s41419-019-1962-x PMC6768886 31570702

[B44] Gu D. Li S. Du M. Tang C. Chu H. Tong N. (2019). A Genetic Variant Located in the miR-532-5p-Binding Site of TGFBR1 Is Associated with the Colorectal Cancer Risk. J. Gastroenterol. 54 (2), 141–148. 10.1007/s00535-018-1490-y 29971498

[B45] Gulei D. Magdo L. Jurj A. Raduly L. Cojocneanu-Petric R. Moldovan A. (2018). The Silent Healer: miR-205-5p Up-Regulation Inhibits Epithelial to Mesenchymal Transition in colon Cancer Cells by Indirectly Up-Regulating E-Cadherin Expression. Cel Death Dis 9 (2), 66. 10.1038/s41419-017-0102-8 PMC5833765 29352232

[B46] Guo H. Liu J. Ben Q. Qu Y. Li M. Wang Y. (2016). The Aspirin-Induced Long Non-coding RNA OLA1P2 Blocks Phosphorylated STAT3 Homodimer Formation. Genome Biol. 17, 24. 10.1186/s13059-016-0892-5 26898989 PMC4762163

[B47] Guo K. Gong W. Wang Q. Gu G. Zheng T. Li Y. (2020). LINC01106 Drives Colorectal Cancer Growth and Stemness through a Positive Feedback Loop to Regulate the Gli Family Factors. Cel Death Dis 11 (10), 869. 10.1038/s41419-020-03026-3 PMC7567881 33067422

[B48] Guo X. Zhu Y. Hong X. Zhang M. Qiu X. Wang Z. (2017). miR-181d and C-Myc-Mediated Inhibition of CRY2 and FBXL3 Reprograms Metabolism in Colorectal Cancer. Cel Death Dis 8 (7), e2958. 10.1038/cddis.2017.300 PMC5550850 28749470

[B49] Guo Y. Ye Q. Deng P. Cao Y. He D. Zhou Z. (2020). Spermine Synthase and MYC Cooperate to Maintain Colorectal Cancer Cell Survival by Repressing Bim Expression. Nat. Commun. 11 (1), 3243. 10.1038/s41467-020-17067-x 32591507 PMC7320137

[B50] Han P. Li J.-w. Zhang B.-m. Lv J.-c. Li Y.-m. Gu X.-y. (2017). The lncRNA CRNDE Promotes Colorectal Cancer Cell Proliferation and Chemoresistance via miR-181a-5p-Mediated Regulation of Wnt/β-Catenin Signaling. Mol. Cancer 16 (1), 9. 10.1186/s12943-017-0583-1 28086904 PMC5237133

[B51] Han Q. Xu L. Lin W. Yao X. Jiang M. Zhou R. (2019). Long Noncoding RNA CRCMSL Suppresses Tumor Invasive and Metastasis in Colorectal Carcinoma through Nucleocytoplasmic Shuttling of HMGB2. Oncogene 38 (16), 3019–3032. 10.1038/s41388-018-0614-4 30575817

[B52] He C. Yu T. Shi Y. Ma C. Yang W. Fang L. (2017). MicroRNA 301A Promotes Intestinal Inflammation and Colitis-Associated Cancer Development by Inhibiting BTG1. Gastroenterology 152 (6), 1434–1448. 10.1053/j.gastro.2017.01.049 28193514

[B53] He F. Song Z. Chen H. Chen Z. Yang P. Li W. (2019). Long Noncoding RNA PVT1-214 Promotes Proliferation and Invasion of Colorectal Cancer by Stabilizing Lin28 and Interacting with miR-128. Oncogene 38 (2), 164–179. 10.1038/s41388-018-0432-8 30076414 PMC6329639

[B54] Hou P. Meng S. Li M. Lin T. Chu S. Li Z. (2021). LINC00460/DHX9/IGF2BP2 Complex Promotes Colorectal Cancer Proliferation and Metastasis by Mediating HMGA1 mRNA Stability Depending on m6A Modification. J. Exp. Clin. Cancer Res. 40 (1), 52. 10.1186/s13046-021-01857-2 33526059 PMC7851923

[B55] Hu J. L. Wang W. Lan X. L. Zeng Z. C. Liang Y. S. Yan Y. R. (2019). CAFs Secreted Exosomes Promote Metastasis and Chemotherapy Resistance by Enhancing Cell Stemness and Epithelial-Mesenchymal Transition in Colorectal Cancer. Mol. Cancer 18 (1), 91. 10.1186/s12943-019-1019-x 31064356 PMC6503554

[B56] Huang H. Weng H. Sun W. Qin X. Shi H. Wu H. (2018). Recognition of RNA N6-Methyladenosine by IGF2BP Proteins Enhances mRNA Stability and Translation. Nat. Cel Biol 20 (3), 285–295. 10.1038/s41556-018-0045-z PMC5826585 29476152

[B57] Huang L. Wen C. Yang X. Lou Q. Wang X. Che J. (2018). PEAK1, Acting as a Tumor Promoter in Colorectal Cancer, Is Regulated by the EGFR/KRas Signaling axis and miR-181d. Cel Death Dis 9 (3), 271. 10.1038/s41419-018-0320-8 PMC5833579 29449544

[B58] Huang Q. Xia J. Wang L. Wang X. Ma X. Deng Q. (2018). miR-153 Suppresses Ido1 Expression and Enhances CAR T Cell Immunotherapy. J. Hematol. Oncol. 11 (1), 58. 10.1186/s13045-018-0600-x 29685162 PMC5914051

[B59] Hübner J.-M. Müller T. Papageorgiou D. N. Mauermann M. Krijgsveld J. Russell R. B. (2019). EZHIP/CXorf67 Mimics K27M Mutated Oncohistones and Functions as an Intrinsic Inhibitor of PRC2 Function in Aggressive Posterior Fossa Ependymoma. Neuro Oncol. 21 (7), 878–889. 10.1093/neuonc/noz058 30923826 PMC6620627

[B60] Jacob J. Favicchio R. Karimian N. Mehrabi M. Harding V. Castellano L. (2016). LMTK3 Escapes Tumour Suppressor miRNAs via Sequestration of DDX5. Cancer Lett. 372 (1), 137–146. 10.1016/j.canlet.2015.12.026 26739063

[B61] Ji D. Zhan T. Li M. Yao Y. Jia J. Yi H. (2018). Enhancement of Sensitivity to Chemo/Radiation Therapy by Using miR-15b against DCLK1 in Colorectal Cancer. Stem Cel Rep. 11 (6), 1506–1522. 10.1016/j.stemcr.2018.10.015 PMC6294114 30449704

[B62] Ji L. Li X. Zhou Z. Zheng Z. Jin L. Jiang F. (2020). LINC01413/hnRNP-K/ZEB1 Axis Accelerates Cell Proliferation and EMT in Colorectal Cancer via Inducing YAP1/TAZ1 Translocation. Mol. Ther. - Nucleic Acids 19, 546–561. 10.1016/j.omtn.2019.11.027 31927328 PMC6953771

[B63] Ji Q. Cai G. Liu X. Zhang Y. Wang Y. Zhou L. (2019). MALAT1 Regulates the Transcriptional and Translational Levels of Proto-Oncogene RUNX2 in Colorectal Cancer Metastasis. Cel Death Dis 10 (6), 378. 10.1038/s41419-019-1598-x PMC6522477 31097689

[B64] Jiang B. Tang Y. Wang H. Chen C. Yu W. Sun H. (2020). Down-regulation of Long Non-coding RNA HOTAIR Promotes Angiogenesis via Regulating miR-126/SCEL Pathways in Burn Wound Healing. Cel Death Dis 11 (1), 61. 10.1038/s41419-020-2247-0 PMC6978466 31974341

[B65] Jiang H. Li T. Qu Y. Wang X. Li B. Song J. (2018). Long Non-coding RNA SNHG15 Interacts with and Stabilizes Transcription Factor Slug and Promotes colon Cancer Progression. Cancer Lett. 425, 78–87. 10.1016/j.canlet.2018.03.038 29604394

[B66] Jiang H. Wang Y. Ai M. Wang H. Duan Z. Wang H. (2017). Long Noncoding RNA CRNDE Stabilized by hnRNPUL2 Accelerates Cell Proliferation and Migration in Colorectal Carcinoma via Activating Ras/MAPK Signaling Pathways. Cel Death Dis 8 (6), e2862. 10.1038/cddis.2017.258 PMC5520914 28594403

[B67] Jiang L. Hermeking H. (2017). miR-34a and miR-34b/c Suppress Intestinal Tumorigenesis. Cancer Res. 77 (10), 2746–2758. 10.1158/0008-5472.can-16-2183 28363996

[B68] Jiang M. Xu B. Li X. Shang Y. Chu Y. Wang W. (2019). O-GlcNAcylation Promotes Colorectal Cancer Metastasis via the miR-101-O-GlcNAc/EZH2 Regulatory Feedback Circuit. Oncogene 38 (3), 301–316. 10.1038/s41388-018-0435-5 30093632 PMC6336687

[B69] Jin F. Yang R. Wei Y. Wang D. Zhu Y. Wang X. (2019). HIF-1α-induced miR-23a∼27a∼24 Cluster Promotes Colorectal Cancer Progression via Reprogramming Metabolism. Cancer Lett. 440-441, 211–222. 10.1016/j.canlet.2018.10.025 30393198

[B70] Kawasaki Y. Miyamoto M. Oda T. Matsumura K. Negishi L. Nakato R. (2019). The Novel lncRNA CALIC Upregulates AXL to Promote colon Cancer Metastasis. EMBO Rep. 20 (8), e47052. 10.15252/embr.201847052 31353791 PMC6680119

[B71] Kawasaki Y. Komiya M. Matsumura K. Negishi L. Suda S. Okuno M. (2016). MYU, a Target lncRNA for Wnt/c-Myc Signaling, Mediates Induction of CDK6 to Promote Cell Cycle Progression. Cel Rep. 16 (10), 2554–2564. 10.1016/j.celrep.2016.08.015 27568568

[B72] Kelley K. A. Ruhl R. A. Rana S. R. Dewey E. Espinosa C. Thomas C. R. Jr. (2017). Understanding and Resetting Radiation Sensitivity in Rectal Cancer. Ann. Surg. 266 (4), 610–616. 10.1097/sla.0000000000002409 28742699 PMC7289057

[B73] Kheradmand Kia S. Solaimani Kartalaei P. Farahbakhshian E. Pourfarzad F. von Lindern M. Verrijzer C. P. (2009). EZH2-dependent Chromatin Looping Controls INK4a and INK4b, but Not ARF, during Human Progenitor Cell Differentiation and Cellular Senescence. Epigenetics & Chromatin 2 (1), 16. 10.1186/1756-8935-2-16 19954516 PMC3225837

[B74] Kim C. Hong Y. Lee H. Kang H. Lee E. K. (2018). MicroRNA-195 Desensitizes HCT116 Human colon Cancer Cells to 5-fluorouracil. Cancer Lett. 412, 264–271. 10.1016/j.canlet.2017.10.022 29080751

[B75] Kim C. W. Oh E.-T. Kim J. M. Park J.-S. Lee D. H. Lee J.-S. (2019). Hypoxia-induced microRNA-590-5p Promotes Colorectal Cancer Progression by Modulating Matrix Metalloproteinase Activity. Cancer Lett. 416, 3173–3241. 10.1016/j.canlet.2017.12.018 29247825

[B76] Kim Y.-J. Lee J. H. Jin S. Kim J. H. Kim S. H. (2019). Primate-specific miR-944 Activates P53-dependent Tumor Suppression in Human Colorectal Cancers. Cancer Lett. 440-441, 168–179. 10.1016/j.canlet.2018.10.029 30393117

[B77] Kong J. Sun W. Li C. Wan L. Wang S. Wu Y. (2016). Long Non-coding RNA LINC01133 Inhibits Epithelial-Mesenchymal Transition and Metastasis in Colorectal Cancer by Interacting with SRSF6. Cancer Lett. 380 (2), 476–484. 10.1016/j.canlet.2016.07.015 27443606

[B78] Kristensen L. S. Ebbesen K. K. Sokol M. Jakobsen T. Korsgaard U. Eriksen A. C. (2020). Spatial Expression Analyses of the Putative Oncogene ciRS-7 in Cancer Reshape the microRNA Sponge Theory. Nat. Commun. 11 (1), 4551. 10.1038/s41467-020-18355-2 32917870 PMC7486402

[B79] Lee Y. S. Dutta A. (2009). MicroRNAs in Cancer. Annu. Rev. Pathol. Mech. Dis. 4, 199–227. 10.1146/annurev.pathol.4.110807.092222 PMC2769253 18817506

[B80] Li H. Rokavec M. Jiang L. Horst D. Hermeking H. (2017). Antagonistic Effects of P53 and HIF1A on microRNA-34a Regulation of PPP1R11 and STAT3 and Hypoxia-Induced Epithelial to Mesenchymal Transition in Colorectal Cancer Cells. Gastroenterology 153 (2), 505–520. 10.1053/j.gastro.2017.04.017 28435028

[B81] Li J. Song P. Jiang T. Dai D. Wang H. Sun J. (2018). Heat Shock Factor 1 Epigenetically Stimulates Glutaminase-1-dependent mTOR Activation to Promote Colorectal Carcinogenesis. Mol. Ther. 26 (7), 1828–1839. 10.1016/j.ymthe.2018.04.014 29730197 PMC6035735

[B82] Li J. Sun D. Pu W. Wang J. Peng Y. (2020). Circular RNAs in Cancer: Biogenesis, Function, and Clinical Significance. Trends Cancer 6 (4), 319–336. 10.1016/j.trecan.2020.01.012 32209446

[B83] Li L. Kang L. Zhao W. Feng Y. Liu W. Wang T. (2017). miR-30a-5p Suppresses Breast Tumor Growth and Metastasis through Inhibition of LDHA-Mediated Warburg Effect. Cancer Lett. 400, 89–98. 10.1016/j.canlet.2017.04.034 28461244

[B84] Li P. Zhang X. Wang L. Du L. Yang Y. Liu T. (2017). lncRNA HOTAIR Contributes to 5FU Resistance through Suppressing miR-218 and Activating NF-Κb/TS Signaling in Colorectal Cancer. Mol. Ther. - Nucleic Acids 8, 356–369. 10.1016/j.omtn.2017.07.007 28918035 PMC5537205

[B85] Li Q. Wang Y. Wu S. Zhou Z. Ding X. Shi R. (2019). CircACC1 Regulates Assembly and Activation of AMPK Complex under Metabolic Stress. Cel Metab. 30 (1), 157–173. 10.1016/j.cmet.2019.05.009 31155494

[B86] Li Y. Sun Z. Liu B. Shan Y. Zhao L. Jia L. (2017). Tumor-suppressive miR-26a and miR-26b Inhibit Cell Aggressiveness by Regulating FUT4 in Colorectal Cancer. Cel Death Dis 8 (6), e2892. 10.1038/cddis.2017.281 PMC5520934 28640257

[B87] Li Y. Zeng C. Hu J. Pan Y. Shan Y. Liu B. (2018). Long Non-coding RNA-SNHG7 Acts as a Target of miR-34a to Increase GALNT7 Level and Regulate PI3K/Akt/mTOR Pathway in Colorectal Cancer Progression. J. Hematol. Oncol. 11 (1), 89. 10.1186/s13045-018-0632-2 29970122 PMC6029165

[B88] Lian Y. Yan C. Xu H. Yang J. Yu Y. Zhou J. (2018). A Novel lncRNA, LINC00460, Affects Cell Proliferation and Apoptosis by Regulating KLF2 and CUL4A Expression in Colorectal Cancer. Mol. Ther. - Nucleic Acids 12, 684–697. 10.1016/j.omtn.2018.06.012 30092404 PMC6083012

[B89] Liang C. Zhao T. Li H. He F. Zhao X. Zhang Y. (2019). Long Non-coding RNA ITIH4-AS1 Accelerates the Proliferation and Metastasis of Colorectal Cancer by Activating JAK/STAT3 Signaling. Mol. Ther. - Nucleic Acids 18, 183–193. 10.1016/j.omtn.2019.08.009 31557619 PMC6796638

[B90] Liang G. Zhu Y. Ali D. J. Tian T. Xu H. Si K. (2020). Engineered Exosomes for Targeted Co-delivery of miR-21 Inhibitor and Chemotherapeutics to Reverse Drug Resistance in colon Cancer. J. Nanobiotechnol 18 (1), 10. 10.1186/s12951-019-0563-2 PMC6950820 31918721

[B91] Liang L. Gao C. Li Y. Sun M. Xu J. Li H. (2017). miR-125a-3p/FUT5-FUT6 axis Mediates Colorectal Cancer Cell Proliferation, Migration, Invasion and Pathological Angiogenesis via PI3K-Akt Pathway. Cel Death Dis 8 (8), e2968. 10.1038/cddis.2017.352 PMC5596543 28771224

[B92] Liang Y. Hou L. Li L. Li L. Zhu L. Wang Y. (2020). Dichloroacetate Restores Colorectal Cancer Chemosensitivity through the p53/miR-149-3p/PDK2-Mediated Glucose Metabolic Pathway. Oncogene 39 (2), 469–485. 10.1038/s41388-019-1035-8 31597953 PMC6949190

[B93] Liang Z.-x. Liu H.-s. Wang F.-w. Xiong L. Zhou C. Hu T. (2019). LncRNA RPPH1 Promotes Colorectal Cancer Metastasis by Interacting with TUBB3 and by Promoting Exosomes-Mediated Macrophage M2 Polarization. Cel Death Dis 10 (11), 829. 10.1038/s41419-019-2077-0 PMC6828701 31685807

[B94] Lin C. Zhang Y. Chen Y. Bai Y. Zhang Y. (2019). Long Noncoding RNA LINC01234 Promotes Serine Hydroxymethyltransferase 2 Expression and Proliferation by Competitively Binding miR-642a-5p in colon Cancer. Cel Death Dis 10 (2), 137. 10.1038/s41419-019-1352-4 PMC6372696 30755591

[B95] Lin J. Tan X. Qiu L. Huang L. Zhou Y. Pan Z. (2017). Long Noncoding RNA BC032913 as a Novel Therapeutic Target for Colorectal Cancer that Suppresses Metastasis by Upregulating TIMP3. Mol. Ther. - Nucleic Acids 8, 469–481. 10.1016/j.omtn.2017.07.009 28918047 PMC5545770

[B96] Lin X. Wang S. Sun M. Zhang C. Wei C. Yang C. (2019). miR-195-5p/NOTCH2-mediated EMT Modulates IL-4 Secretion in Colorectal Cancer to Affect M2-like TAM Polarization. J. Hematol. Oncol. 12 (1), 20. 10.1186/s13045-019-0708-7 30808369 PMC6390326

[B97] Ling H. Pickard K. Ivan C. Isella C. Ikuo M. Mitter R. (2016). The Clinical and Biological Significance of MIR-224 Expression in Colorectal Cancer Metastasis. Gut 65 (6), 977–989. 10.1136/gutjnl-2015-309372 25804630 PMC4581915

[B98] Liu J. Zhan Y. Wang J. Wang J. Guo J. Kong D. (2020). Long Noncoding RNA LINC01578 Drives colon Cancer Metastasis through a Positive Feedback Loop with the NF‐κB/YY1 axis. Mol. Oncol. 14 (12), 3211–3233. 10.1002/1878-0261.12819 33040438 PMC7718957

[B99] Liu K. Chen W. Lei S. Xiong L. Zhao H. Liang D. (2017). Wild-type and Mutant P53 Differentially Modulate miR-124/iASPP Feedback Following Pohotodynamic Therapy in Human colon Cancer Cell Line. Cel Death Dis 8 (10), e3096. 10.1038/cddis.2017.477 PMC5682646 29022915

[B100] Liu L. Wang H.-J. Meng T. Lei C. Yang X.-H. Wang Q.-S. (2019). lncRNA GAS5 Inhibits Cell Migration and Invasion and Promotes Autophagy by Targeting miR-222-3p via the GAS5/PTEN-Signaling Pathway in CRC. Mol. Ther. - Nucleic Acids 17, 644–656. 10.1016/j.omtn.2019.06.009 31400607 PMC6698928

[B101] Liu R. Zhang Q. Shen L. Chen S. He J. Wang D. (2020). Long Noncoding RNA Lnc-RI Regulates DNA Damage Repair and Radiation Sensitivity of CRC Cells through NHEJ Pathway. Cell Biol Toxicol 36 (5), 493–507. 10.1007/s10565-020-09524-6 32279126

[B102] Liu T. Zhang X. Du L. Wang Y. Liu X. Tian H. (2019). Exosome-transmitted miR-128-3p Increase Chemosensitivity of Oxaliplatin-Resistant Colorectal Cancer. Mol. Cancer 18 (1), 43. 10.1186/s12943-019-0981-7 30890168 PMC6423768

[B103] Liu X. Chen X. Zeng K. Xu M. He B. Pan Y. (2018). DNA-methylation-mediated Silencing of miR-486-5p Promotes Colorectal Cancer Proliferation and Migration through Activation of PLAGL2/IGF2/β-Catenin Signal Pathways. Cel Death Dis 9 (10), 1037. 10.1038/s41419-018-1105-9 PMC6180105 30305607

[B104] Liu X. Su K. Sun X. Jiang Y. Wang L. Hu C. (2021). Sec62 Promotes Stemness and Chemoresistance of Human Colorectal Cancer through Activating Wnt/β-Catenin Pathway. J. Exp. Clin. Cancer Res. 40 (1), 132. 10.1186/s13046-021-01934-6 33858476 PMC8051072

[B105] Liu Y. Chen X. Chen X. Liu J. Gu H. Fan R. (2020). Long Non-coding RNA HOTAIR Knockdown Enhances Radiosensitivity through Regulating microRNA-93/ATG12 axis in Colorectal Cancer. Cel Death Dis 11 (3), 175. 10.1038/s41419-020-2268-8 PMC7060216 32144238

[B106] Liu Y. Chen X. Cheng R. Yang F. Yu M. Wang C. (2018). The Jun/miR-22/HuR Regulatory axis Contributes to Tumourigenesis in Colorectal Cancer. Mol. Cancer 17 (1), 11. 10.1186/s12943-017-0751-3 29351796 PMC5775639

[B107] Liu Y. Liu R. Yang F. Cheng R. Chen X. Cui S. (2017). miR-19a Promotes Colorectal Cancer Proliferation and Migration by Targeting TIA1. Mol. Cancer 16 (1), 53. 10.1186/s12943-017-0625-8 28257633 PMC5336638

[B108] Liu Y. Uzair-ur-Rehman R. Guo Y. Liang H. Cheng R. Yang F. (2016). miR-181b Functions as an oncomiR in Colorectal Cancer by Targeting PDCD4. Protein Cell 7 (10), 722–734. 10.1007/s13238-016-0313-2 27647131 PMC5055492

[B109] Liu Y. Zhang Y. Wu H. Li Y. Zhang Y. Liu M. (2017). miR-10a Suppresses Colorectal Cancer Metastasis by Modulating the Epithelial-To-Mesenchymal Transition and Anoikis. Cel Death Dis 8 (4), e2739. 10.1038/cddis.2017.61 PMC5477594 28383561

[B110] Long F. Lin Z. Li L. Ma M. Lu Z. Jing L. (2021). Comprehensive Landscape and Future Perspectives of Circular RNAs in Colorectal Cancer. Mol. Cancer 20 (1), 26. 10.1186/s12943-021-01318-6 33536039 PMC7856739

[B111] Lou Q. Liu R. Yang X. Li W. Huang L. Wei L. (2019). miR-448 Targets Ido1 and Regulates CD8+ T Cell Response in Human colon Cancer. J. Immunotherapy Cancer 7 (1), 210. 10.1186/s40425-019-0691-0 PMC6686234 31391111

[B112] Lu Y. Zhao X. Liu Q. Li C. Graves-Deal R. Cao Z. (2017). lncRNA MIR100HG-Derived miR-100 and miR-125b Mediate Cetuximab Resistance via Wnt/β-Catenin Signaling. Nat. Med. 23 (11), 1331–1341. 10.1038/nm.4424 29035371 PMC5961502

[B113] Luan Y. Li X. Luan Y. Zhao R. Li Y. Liu L. (2020). Circulating lncRNA UCA1 Promotes Malignancy of Colorectal Cancer via the miR-143/MYO6 Axis. Mol. Ther. - Nucleic Acids 19, 790–803. 10.1016/j.omtn.2019.12.009 31955010 PMC6970172

[B114] Lulla A. R. Slifker M. J. Zhou Y. Lev A. Einarson M. B. Dicker D. T. (2017). miR-6883 Family miRNAs Target CDK4/6 to Induce G1 Phase Cell-Cycle Arrest in Colon Cancer Cells. Cancer Res. 77 (24), 6902–6913. 10.1158/0008-5472.can-17-1767 29061672

[B115] Luo H. Jiang Y. Ma S. Chang H. Yi C. Cao H. (2016). EZH2 Promotes Invasion and Metastasis of Laryngeal Squamous Cells Carcinoma via Epithelial-Mesenchymal Transition through H3K27me3. Biochem. Biophysical Res. Commun. 479 (2), 253–259. 10.1016/j.bbrc.2016.09.055 27638307

[B116] Luo Y. Chen J.-J. Lv Q. Qin J. Huang Y.-Z. Yu M.-H. (2019). Long Non-coding RNA NEAT1 Promotes Colorectal Cancer Progression by Competitively Binding miR-34a with SIRT1 and Enhancing the Wnt/β-Catenin Signaling Pathway. Cancer Lett. 440-441, 11–22. 10.1016/j.canlet.2018.10.002 30312725

[B117] Ma H. Pan J.-S. Jin L.-X. Wu J. Ren Y.-D. Chen P. (2016). MicroRNA-17∼92 Inhibits Colorectal Cancer Progression by Targeting Angiogenesis. Cancer Lett. 376 (2), 293–302. 10.1016/j.canlet.2016.04.011 27080303

[B118] Ma J. Yang Y. Fu Y. Guo F. Zhang X. Xiao S. (2018). PIAS3-mediated Feedback Loops Promote Chronic Colitis-Associated Malignant Transformation. Theranostics 8 (11), 3022–3037. 10.7150/thno.23046 29896300 PMC5996365

[B119] Ma Y. Yang Y. Wang F. Moyer M.-P. Wei Q. Zhang P. (2016). Long Non-coding RNA CCAL Regulates Colorectal Cancer Progression by Activating Wnt/β-Catenin Signalling Pathway via Suppression of Activator Protein 2α. Gut 65 (9), 1494–1504. 10.1136/gutjnl-2014-308392 25994219

[B120] Ma Z. Han C. Xia W. Wang S. Li X. Fang P. (2020). circ5615 Functions as a ceRNA to Promote Colorectal Cancer Progression by Upregulating TNKS. Cel Death Dis 11 (5), 356. 10.1038/s41419-020-2514-0 PMC7214456 32393760

[B121] Mai D. Ding P. Tan L. Zhang J. Pan Z. Bai R. (2018). PIWI-interacting RNA-54265 Is Oncogenic and a Potential Therapeutic Target in Colorectal Adenocarcinoma. Theranostics 8 (19), 5213–5230. 10.7150/thno.28001 30555542 PMC6276099

[B122] Mastropasqua F. Marzano F. Valletti A. Aiello I. Di Tullio G. Morgano A. (2017). TRIM8 Restores P53 Tumour Suppressor Function by Blunting N-MYC Activity in Chemo-Resistant Tumours. Mol. Cancer 16 (1), 67. 10.1186/s12943-017-0634-7 28327152 PMC5359838

[B123] Matsumura K. Kawasaki Y. Miyamoto M. Kamoshida Y. Nakamura J. Negishi L. (2017). The Novel G-Quadruplex-Containing Long Non-coding RNA GSEC Antagonizes DHX36 and Modulates colon Cancer Cell Migration. Oncogene 36 (9), 1191–1199. 10.1038/onc.2016.282 27797375

[B124] McCabe M. T. Graves A. P. Ganji G. Diaz E. Halsey W. S. Jiang Y. (2012). Mutation of A677 in Histone Methyltransferase EZH2 in Human B-Cell Lymphoma Promotes Hypertrimethylation of Histone H3 on Lysine 27 (H3K27). Proc. Natl. Acad. Sci. 109 (8), 2989–2994. 10.1073/pnas.1116418109 22323599 PMC3287005

[B125] Meng X. Sun W. Yu J. Zhou Y. Gu Y. Han J. (2020). LINC00460-miR-149-5p/miR-150-5p-Mutant P53 Feedback Loop Promotes Oxaliplatin Resistance in Colorectal Cancer. Mol. Ther. - Nucleic Acids 22, 1004–1015. 10.1016/j.omtn.2020.10.018 33251049 PMC7679243

[B126] Michael J. V. Wurtzel J. G. T. Mao G. F. Rao A. K. Kolpakov M. A. Sabri A. (2017). Platelet Microparticles Infiltrating Solid Tumors Transfer miRNAs that Suppress Tumor Growth. Blood 130 (5), 567–580. 10.1182/blood-2016-11-751099 28500171 PMC5542851

[B127] Mo S. Zhang L. Dai W. Han L. Wang R. Xiang W. (2020). Antisense lncRNA LDLRAD4-AS1 Promotes Metastasis by Decreasing the Expression of LDLRAD4 and Predicts a Poor Prognosis in Colorectal Cancer. Cel Death Dis 11 (2), 155. 10.1038/s41419-020-2338-y PMC7048743 32111819

[B128] Mosthaf F. A. Gieseler U. Mehmel H. C. Fischer J. T. Gams E. (1991). Left Bundle-branch Block and Primary Benign Heart Tumor. Dtsch Med. Wochenschr 116 (4), 134–136. 10.1055/s-2008-1063592 1988275

[B129] Mukohyama J. Isobe T. Hu Q. Hayashi T. Watanabe T. Maeda M. (2019). miR-221 Targets QKI to Enhance the Tumorigenic Capacity of Human Colorectal Cancer Stem Cells. Cancer Res. 79 (20), 5151–5158. 10.1158/0008-5472.can-18-3544 31416845 PMC6801097

[B130] Ni W. Yao S. Zhou Y. Liu Y. Huang P. Zhou A. (2019). Long Noncoding RNA GAS5 Inhibits Progression of Colorectal Cancer by Interacting with and Triggering YAP Phosphorylation and Degradation and Is Negatively Regulated by the m6A Reader YTHDF3. Mol. Cancer 18 (1), 143. 10.1186/s12943-019-1079-y 31619268 PMC6794841

[B131] Okugawa Y. Toiyama Y. Hur K. Yamamoto A. Yin C. Ide S. (2019). Circulating miR‐203 Derived from Metastatic Tissues Promotes Myopenia in Colorectal Cancer Patients. J. Cachexia, Sarcopenia Muscle 10 (3), 536–548. 10.1002/jcsm.12403 31091026 PMC6596405

[B132] Öner M. G. Rokavec M. Kaller M. Bouznad N. Horst D. Kirchner T. (2018). Combined Inactivation of TP53 and MIR34A Promotes Colorectal Cancer Development and Progression in Mice via Increasing Levels of IL6R and PAI1. Gastroenterology 155 (6), 1868–1882. 10.1053/j.gastro.2018.08.011 30099074

[B133] Oshima G. Guo N. He C. Stack M. E. Poon C. Uppal A. (2017). *In Vivo* Delivery and Therapeutic Effects of a MicroRNA on Colorectal Liver Metastases. Mol. Ther. 25 (7), 1588–1595. 10.1016/j.ymthe.2017.04.005 28457664 PMC5498809

[B134] Ou C. Sun Z. Li X. Li X. Ren W. Qin Z. (2018). MiR-590-5p, a Density-Sensitive microRNA, Inhibits Tumorigenesis by Targeting YAP1 in Colorectal Cancer. Cancer Lett. 399, 53260–53263. 10.1016/j.canlet.2017.04.011 28433598

[B135] Pan K. Xie Y. (2020). LncRNA FOXC2-AS1 Enhances FOXC2 mRNA Stability to Promote Colorectal Cancer Progression via Activation of Ca2+-FAK Signal Pathway. Cel Death Dis 11 (6), 434. 10.1038/s41419-020-2633-7 PMC7280533 32513911

[B136] Pekow J. Meckel K. Dougherty U. Huang Y. Chen X. Almoghrabi A. (2017). miR-193a-3p Is a Key Tumor Suppressor in Ulcerative Colitis-Associated Colon Cancer and Promotes Carcinogenesis through Upregulation of IL17RD. Clin. Cancer Res. 23 (17), 5281–5291. 10.1158/1078-0432.ccr-17-0171 28600480 PMC5581687

[B137] Peng W. Zhang C. Peng J. Huang Y. Peng C. Tan Y. (2020). Lnc-FAM84B-4 Acts as an Oncogenic lncRNA by Interacting with Protein hnRNPK to Restrain MAPK Phosphatases-DUSP1 Expression. Cancer Lett. 494, 94–106. 10.1016/j.canlet.2020.08.036 32866608

[B138] Pichler M. Rodriguez-Aguayo C. Nam S. Y. Dragomir M. P. Bayraktar R. Anfossi S. (2020). Therapeutic Potential of FLANC, a Novel Primate-specific Long Non-coding RNA in Colorectal Cancer. Gut 69 (10), 1818–1831. 10.1136/gutjnl-2019-318903 31988194 PMC7382985

[B139] Rasmussen M. H. Lyskjær I. Jersie-Christensen R. R. Tarpgaard L. S. Primdal-Bengtson B. Nielsen M. M. (2016). miR-625-3p Regulates Oxaliplatin Resistance by Targeting MAP2K6-P38 Signalling in Human Colorectal Adenocarcinoma Cells. Nat. Commun. 7, 12436. 10.1038/ncomms12436 27526785 PMC4990699

[B140] Ren J. Ding L. Zhang D. Shi G. Xu Q. Shen S. (2018). Carcinoma-associated Fibroblasts Promote the Stemness and Chemoresistance of Colorectal Cancer by Transferring Exosomal lncRNA H19. Theranostics 8 (14), 3932–3948. 10.7150/thno.25541 30083271 PMC6071523

[B141] Ren L.-L. Yan T.-T. Shen C.-Q. Tang J.-Y. Kong X. Wang Y.-C. (2018). The Distinct Role of Strand-specific miR-514b-3p and miR-514b-5p in Colorectal Cancer Metastasis. Cel Death Dis 9 (6), 687. 10.1038/s41419-018-0732-5 PMC5992212 29880874

[B142] Rigoutsos I. Lee S. K. Nam S. Y. Anfossi S. Pasculli B. Pichler M. (2017). N-BLR, a Primate-specific Non-coding Transcript Leads to Colorectal Cancer Invasion and Migration. Genome Biol. 18 (1), 98. 10.1186/s13059-017-1224-0 28535802 PMC5442648

[B143] Rokavec M. Bouznad N. Hermeking H. (2019). Paracrine Induction of Epithelial-Mesenchymal Transition between Colorectal Cancer Cells and its Suppression by a p53/miR-192/215/NID1 Axis. Cell Mol. Gastroenterol. Hepatol. 7 (4), 783–802. 10.1016/j.jcmgh.2019.02.003 30831320 PMC6468198

[B144] Sang B. Zhang Y. Y. Guo S. T. Kong L. F. Cheng Q. Liu G. Z. (2018). Dual Functions for OVAAL in Initiation of RAF/MEK/ERK Prosurvival Signals and Evasion of P27-Mediated Cellular Senescence. Proc. Natl. Acad. Sci. USA 115 (50), E11661–E11670. 10.1073/pnas.1805950115 30478051 PMC6294934

[B145] Shan Y. Ma J. Pan Y. Hu J. Liu B. Jia L. (2018). LncRNA SNHG7 Sponges miR-216b to Promote Proliferation and Liver Metastasis of Colorectal Cancer through Upregulating GALNT1. Cel Death Dis 9 (7), 722. 10.1038/s41419-018-0759-7 PMC6006356 29915311

[B146] Shang W. Gao Y. Tang Z. Zhang Y. Yang R. (2019). The Pseudogene Olfr29-Ps1 Promotes the Suppressive Function and Differentiation of Monocytic MDSCs. Cancer Immunol. Res. 7 (5), 813–827. 10.1158/2326-6066.cir-18-0443 30914411

[B147] Shen H. Xing C. Cui K. Li Y. Zhang J. Du R. (2017). MicroRNA-30a Attenuates Mutant KRAS-Driven Colorectal Tumorigenesis via Direct Suppression of ME1. Cell Death Differ 24 (7), 1253–1262. 10.1038/cdd.2017.63 28475173 PMC5520171

[B148] Shen Z. Zhou R. Liu C. Wang Y. Zhan W. Shao Z. (2017). MicroRNA-105 Is Involved in TNF-α-Related Tumor Microenvironment Enhanced Colorectal Cancer Progression. Cel Death Dis 8 (12), 3213. 10.1038/s41419-017-0048-x PMC5870598 29238068

[B149] Shi C. Yang Y. Xia Y. Okugawa Y. Yang J. Liang Y. (2016). Novel Evidence for an Oncogenic Role of microRNA-21 in Colitis-Associated Colorectal Cancer. Gut 65 (9), 1470–1481. 10.1136/gutjnl-2014-308455 25994220

[B150] Shi L. Hong X. Ba L. He X. Xiong Y. Ding Q. (2019). Long Non-coding RNA ZNFX1-AS1 Promotes the Tumor Progression and Metastasis of Colorectal Cancer by Acting as a Competing Endogenous RNA of miR-144 to Regulate EZH2 Expression. Cel Death Dis 10 (3), 150. 10.1038/s41419-019-1332-8 PMC6377660 30770796

[B151] Shigeyasu K. Toden S. Ozawa T. Matsuyama T. Nagasaka T. Ishikawa T. (2020). The PVT1 lncRNA Is a Novel Epigenetic Enhancer of MYC, and a Promising Risk-Stratification Biomarker in Colorectal Cancer. Mol. Cancer 19 (1), 155. 10.1186/s12943-020-01277-4 33148262 PMC7643275

[B152] Siemens H. Jackstadt R. Kaller M. Hermeking H. (2013). Repression of C-Kit by P53 Is Mediated by miR-34 and Is Associated with Reduced Chemoresistance, Migration and Stemness. Oncotarget 4 (9), 1399–1415. 10.18632/oncotarget.1202 24009080 PMC3824539

[B153] Silva-Fisher J. M. Dang H. X. White N. M. Strand M. S. Krasnick B. A. Rozycki E. B. (2020). Long Non-coding RNA RAMS11 Promotes Metastatic Colorectal Cancer Progression. Nat. Commun. 11 (1), 2156. 10.1038/s41467-020-15547-8 32358485 PMC7195452

[B154] Stiegelbauer V. Vychytilova-Faltejskova P. Karbiener M. Pehserl A.-M. Reicher A. Resel M. (2017). miR-196b-5p Regulates Colorectal Cancer Cell Migration and Metastases through Interaction with HOXB7 and GALNT5. Clin. Cancer Res. 23 (17), 5255–5266. 10.1158/1078-0432.ccr-17-0023 28533224

[B155] Su J. Zhang E. Han L. Yin D. Liu Z. He X. (2017). Long Noncoding RNA BLACAT1 Indicates a Poor Prognosis of Colorectal Cancer and Affects Cell Proliferation by Epigenetically Silencing of P15. Cel Death Dis 8 (3), e2665. 10.1038/cddis.2017.83 PMC5386575 28277544

[B156] Sun L. Fang Y. Wang X. Han Y. Du F. Li C. (2019). miR-302a Inhibits Metastasis and Cetuximab Resistance in Colorectal Cancer by Targeting NFIB and CD44. Theranostics 9 (26), 8409–8425. 10.7150/thno.36605 31754405 PMC6857048

[B157] Sun M. Song H. Wang S. Zhang C. Zheng L. Chen F. (2017). Integrated Analysis Identifies microRNA-195 as a Suppressor of Hippo-YAP Pathway in Colorectal Cancer. J. Hematol. Oncol. 10 (1), 79. 10.1186/s13045-017-0445-8 28356122 PMC5372308

[B158] Sun W. Wang X. Li J. You C. Lu P. Feng H. (2018). MicroRNA-181a Promotes Angiogenesis in Colorectal Cancer by Targeting SRCIN1 to Promote the SRC/VEGF Signaling Pathway. Cel Death Dis 9 (4), 438. 10.1038/s41419-018-0490-4 PMC5941226 29739921

[B159] Sun Z. Ou C. Liu J. Chen C. Zhou Q. Yang S. (2019). YAP1-induced MALAT1 Promotes Epithelial-Mesenchymal Transition and Angiogenesis by Sponging miR-126-5p in Colorectal Cancer. Oncogene 38 (14), 2627–2644. 10.1038/s41388-018-0628-y 30531836 PMC6484768

[B160] Tang J. Yan T. Bao Y. Shen C. Yu C. Zhu X. (2019). LncRNA GLCC1 Promotes Colorectal Carcinogenesis and Glucose Metabolism by Stabilizing C-Myc. Nat. Commun. 10 (1), 3499. 10.1038/s41467-019-11447-8 31375671 PMC6677832

[B161] Tang W. Zhou W. Xiang L. Wu X. Zhang P. Wang J. (2019). The p300/YY1/miR-500a-5p/HDAC2 Signalling axis Regulates Cell Proliferation in Human Colorectal Cancer. Nat. Commun. 10 (1), 663. 10.1038/s41467-018-08225-3 30737378 PMC6368584

[B162] Taniue K. Kurimoto A. Sugimasa H. Nasu E. Takeda Y. Iwasaki K. (2016). Long Noncoding RNA UPAT Promotes colon Tumorigenesis by Inhibiting Degradation of UHRF1. Proc. Natl. Acad. Sci. USA 113 (5), 1273–1278. 10.1073/pnas.1500992113 26768845 PMC4747749

[B163] Taniue K. Kurimoto A. Takeda Y. Nagashima T. Okada-Hatakeyama M. Katou Y. (2016). ASBEL-TCF3 Complex Is Required for the Tumorigenicity of Colorectal Cancer Cells. Proc. Natl. Acad. Sci. USA 113 (45), 12739–12744. 10.1073/pnas.1605938113 27791078 PMC5111701

[B164] Teng Y. Ren Y. Hu X. Mu J. Samykutty A. Zhuang X. (2017). MVP-mediated Exosomal Sorting of miR-193a Promotes colon Cancer Progression. Nat. Commun. 8, 14448. 10.1038/ncomms14448 28211508 PMC5321731

[B165] Thomas H. (2017). miR-100 and miR-125b Induce Cetuximab Resistance in CRC. Nat. Rev. Gastroenterol. Hepatol. 14 (12), 691. 10.1038/nrgastro.2017.156 29094721

[B166] Tian W. Du Y. Ma Y. Gu L. Zhou J. Deng D. (2018). MALAT1-miR663a Negative Feedback Loop in colon Cancer Cell Functions through Direct miRNA-lncRNA Binding. Cel Death Dis 9 (9), 857. 10.1038/s41419-018-0925-y PMC6113222 30154407

[B167] Tian X. Zheng Y. Yin K. Ma J. Tian J. Zhang Y. (2020). LncRNAAK036396Inhibits Maturation and Accelerates Immunosuppression of Polymorphonuclear Myeloid-Derived Suppressor Cells by Enhancing the Stability of Ficolin B. Cancer Immunol. Res. 8 (4), 565–577. 10.1158/2326-6066.cir-19-0595 32102837

[B168] Tian Y. Ma X. Lv C. Sheng X. Li X. Zhao R. (2017). Stress Responsive miR-31 Is a Major Modulator of Mouse Intestinal Stem Cells during Regeneration and Tumorigenesis. Elife 6. 10.7554/eLife.29538 PMC5584991 28870287

[B169] Tian Y. Xu J. Li Y. Zhao R. Du S. Lv C. (2019). MicroRNA-31 Reduces Inflammatory Signaling and Promotes Regeneration in Colon Epithelium, and Delivery of Mimics in Microspheres Reduces Colitis in Mice. Gastroenterology 156 (8), 2281–2296. 10.1053/j.gastro.2019.02.023 30779922

[B170] Tie G. Yan J. Khair L. Messina J. A. Deng A. Kang J. (2017). Hypercholesterolemia Increases Colorectal Cancer Incidence by Reducing Production of NKT and γδ T Cells from Hematopoietic Stem Cells. Cancer Res. 77 (9), 2351–2362. 10.1158/0008-5472.can-16-1916 28249902 PMC6193767

[B171] Tsai K.-W. Lo Y.-H. Liu H. Yeh C.-Y. Chen Y.-Z. Hsu C.-W. (2018). Linc00659, a Long Noncoding RNA, Acts as Novel Oncogene in Regulating Cancer Cell Growth in Colorectal Cancer. Mol. Cancer 17 (1), 72. 10.1186/s12943-018-0821-1 29523145 PMC5845323

[B172] Ullmann P. Nurmik M. Schmitz M. Rodriguez F. Weiler J. Qureshi-Baig K. (2019). Tumor Suppressor miR-215 Counteracts Hypoxia-Induced colon Cancer Stem Cell Activity. Cancer Lett. 450, 32–41. 10.1016/j.canlet.2019.02.030 30790680

[B173] Viswanathan V. Damle S. Zhang T. Opdenaker L. Modarai S. Accerbi M. (2017). An miRNA Expression Signature for the Human Colonic Stem Cell Niche Distinguishes Malignant from Normal Epithelia. Cancer Res. 77 (14), 3778–3790. 10.1158/0008-5472.can-16-2388 28487386 PMC13191046

[B174] Wang D. Wang X. Si M. Yang J. Sun S. Wu H. (2020). Exosome-encapsulated miRNAs Contribute to CXCL12/CXCR4-Induced Liver Metastasis of Colorectal Cancer by Enhancing M2 Polarization of Macrophages. Cancer Lett. 474, 36–52. 10.1016/j.canlet.2020.01.005 31931030

[B175] Wang F.-W. Cao C.-H. Han K. Zhao Y.-X. Cai M.-Y. Xiang Z.-C. (2019). APC-activated Long Noncoding RNA Inhibits Colorectal Carcinoma Pathogenesis through Reduction of Exosome Production. J. Clin. Invest. 129 (2), 727–743. 10.1172/jci122478 30511962 PMC6355227

[B176] Wang F. F. Zhang X. J. Yan Y. R. Zhu X. H. Yu J. Ding Y. (2017). FBX8 Is a Metastasis Suppressor Downstream of miR-223 and Targeting mTOR for Degradation in Colorectal Carcinoma. Cancer Lett. 388, 85–95. 10.1016/j.canlet.2016.11.031 27916606

[B177] Wang G. Fu Y. Yang X. Luo X. Wang J. Gong J. (2016). Brg-1 Targeting of Novel miR550a-5p/RNF43/Wnt Signaling axis Regulates Colorectal Cancer Metastasis. Oncogene 35 (5), 651–661. 10.1038/onc.2015.124 25961913

[B178] Wang J. Zhou J. Jiang C. Zheng J. Namba H. Chi P. (2019). LNRRIL 6, a Novel Long Noncoding RNA , Protects Colorectal Cancer Cells by Activating the IL ‐6- STAT 3 Pathway. Mol. Oncol. 13 (11), 2344–2360. 10.1002/1878-0261.12538 31246342 PMC6822249

[B179] Wang L. Bu P. Ai Y. Srinivasan T. Chen H. J. Xiang K. (2016). A Long Non-coding RNA Targets microRNA miR-34a to Regulate colon Cancer Stem Cell Asymmetric Division. Elife 5. 10.7554/eLife.14620 PMC4859802 27077950

[B180] Wang L. Q. Yu P. Li B. Guo Y. H. Liang Z. R. Zheng L. L. (2018). miR‐372 and miR‐373 Enhance the Stemness of Colorectal Cancer Cells by Repressing Differentiation Signaling Pathways. Mol. Oncol. 12 (11), 1949–1964. 10.1002/1878-0261.12376 30171794 PMC6210048

[B181] Wang M. Han D. Yuan Z. Hu H. Zhao Z. Yang R. (2018). Long Non-coding RNA H19 Confers 5-Fu Resistance in Colorectal Cancer by Promoting SIRT1-Mediated Autophagy. Cel Death Dis 9 (12), 1149. 10.1038/s41419-018-1187-4 PMC6242979 30451820

[B182] Wang Q. Feng Y. Peng W. Ji D. Zhang Z. Qian W. (2019). Long Noncoding RNA Linc02023 Regulates PTEN Stability and Suppresses Tumorigenesis of Colorectal Cancer in a PTEN-dependent Pathway. Cancer Lett. 451, 68–78. 10.1016/j.canlet.2019.02.041 30849479

[B183] Wang S. Gan M. Chen C. Zhang Y. Kong J. Zhang H. (2021). Methyl CpG Binding Protein 2 Promotes Colorectal Cancer Metastasis by Regulating N(6) -methyladenosine Methylation through Methyltransferase-like 14. Cancer Sci. 10.1111/cas.15011 PMC8353896 34097350

[B184] Wang T. Xu X. Xu Q. Ren J. Shen S. Fan C. (2017). miR-19a Promotes Colitis-Associated Colorectal Cancer by Regulating Tumor Necrosis Factor Alpha-Induced Protein 3-NF-Κb Feedback Loops. Oncogene 36 (23), 3240–3251. 10.1038/onc.2016.468 27991929

[B185] Wang X. Wang Y. Li L. Xue X. Xie H. Shi H. (2020). A lncRNA Coordinates with Ezh2 to Inhibit HIF-1α Transcription and Suppress Cancer Cell Adaption to Hypoxia. Oncogene 39 (9), 1860–1874. 10.1038/s41388-019-1123-9 31784651

[B186] Wang Y.-Q. Jiang D.-M. Hu S.-S. Zhao L. Wang L. Yang M.-H. (2019). SATB2-AS1 Suppresses Colorectal Carcinoma Aggressiveness by Inhibiting SATB2-dependent Snail Transcription and Epithelial-Mesenchymal Transition. Cancer Res. 79 (14), 3542–3556. 10.1158/0008-5472.can-18-2900 30858153

[B187] Wang Y. Lu J.-H. Wu Q.-N. Jin Y. Wang D.-S. Chen Y.-X. (2019). LncRNA LINRIS Stabilizes IGF2BP2 and Promotes the Aerobic Glycolysis in Colorectal Cancer. Mol. Cancer 18 (1), 174. 10.1186/s12943-019-1105-0 31791342 PMC6886219

[B188] Weng W. Liu N. Toiyama Y. Kusunoki M. Nagasaka T. Fujiwara T. (2018). Novel Evidence for a PIWI-Interacting RNA (piRNA) as an Oncogenic Mediator of Disease Progression, and a Potential Prognostic Biomarker in Colorectal Cancer. Mol. Cancer 17 (1), 16. 10.1186/s12943-018-0767-3 29382334 PMC5791351

[B189] Weng W. Okugawa Y. Toden S. Toiyama Y. Kusunoki M. Goel A. (2016). FOXM1 and FOXQ1 Are Promising Prognostic Biomarkers and Novel Targets of Tumor-Suppressive miR-342 in Human Colorectal Cancer. Clin. Cancer Res. 22 (19), 4947–4957. 10.1158/1078-0432.ccr-16-0360 27162244 PMC5435114

[B190] Weng W. Wei Q. Toden S. Yoshida K. Nagasaka T. Fujiwara T. (2017). Circular RNA ciRS-7-A Promising Prognostic Biomarker and a Potential Therapeutic Target in Colorectal Cancer. Clin. Cancer Res. 23 (14), 3918–3928. 10.1158/1078-0432.ccr-16-2541 28174233 PMC5511556

[B191] Wu F. Liu F. Dong L. Yang H. He X. Li L. (2018). miR-1273g Silences MAGEA3/6 to Inhibit Human Colorectal Cancer Cell Growth via Activation of AMPK Signaling. Cancer Lett. 435, 1–9. 10.1016/j.canlet.2018.07.031 30056111

[B192] Wu H. Hu X. Li Y. Chen Q. Sun T. Qiao Y. (2020). LNC473 Regulating APAF1 IRES-dependent Translation via Competitive Sponging miR574 and miR15b: Implications in Colorectal Cancer. Mol. Ther. - Nucleic Acids 21, 764–779. 10.1016/j.omtn.2020.07.009 32784109 PMC7419277

[B193] Wu H. Qin W. Lu S. Wang X. Zhang J. Sun T. (2020). Long Noncoding RNA ZFAS1 Promoting Small Nucleolar RNA-Mediated 2′-O-Methylation via NOP58 Recruitment in Colorectal Cancer. Mol. Cancer 19 (1), 95. 10.1186/s12943-020-01201-w 32443980 PMC7243338

[B194] Wu H. Wei M. Jiang X. Tan J. Xu W. Fan X. (2020). lncRNA PVT1 Promotes Tumorigenesis of Colorectal Cancer by Stabilizing miR-16-5p and Interacting with the VEGFA/VEGFR1/AKT Axis. Mol. Ther. - Nucleic Acids 20, 438–450. 10.1016/j.omtn.2020.03.006 32276209 PMC7139143

[B195] Wu R. Li L. Bai Y. Yu B. Xie C. Wu H. (2020). The Long Noncoding RNA LUCAT1 Promotes Colorectal Cancer Cell Proliferation by Antagonizing Nucleolin to Regulate MYC Expression. Cel Death Dis 11 (10), 908. 10.1038/s41419-020-03095-4 PMC7584667 33097685

[B196] Wu S. Sun H. Wang Y. Yang X. Meng Q. Yang H. (2019). MALAT1 Rs664589 Polymorphism Inhibits Binding to miR-194-5p, Contributing to Colorectal Cancer Risk, Growth, and Metastasis. Cancer Res. 79 (20), 5432–5441. 10.1158/0008-5472.can-19-0773 31311811

[B197] Wu Y. Yang X. Chen Z. Tian L. Jiang G. Chen F. (2019). m6A-induced lncRNA RP11 Triggers the Dissemination of Colorectal Cancer Cells via Upregulation of Zeb1A-Induced lncRNA RP11 Triggers the Dissemination of Colorectal Cancer Cells via Upregulation of Zeb1. Mol. Cancer 18 (1), 87. 10.1186/s12943-019-1014-2 30979372 PMC6461827

[B198] Xiao J. Lv D. Zhou J. Bei Y. Chen T. Hu M. (2017). Therapeutic Inhibition of miR-4260 Suppresses Colorectal Cancer via Targeting MCC and SMAD4. Theranostics 7 (7), 1901–1913. 10.7150/thno.19168 28638476 PMC5479277

[B199] Xie S. Yu X. Li Y. Ma H. Fan S. Chen W. (2018). Upregulation of lncRNA ADAMTS9-AS2 Promotes Salivary Adenoid Cystic Carcinoma Metastasis via PI3K/Akt and MEK/Erk Signaling. Mol. Ther. 26 (12), 2766–2778. 10.1016/j.ymthe.2018.08.018 30217729 PMC6277603

[B200] Xu J. Meng Q. Li X. Yang H. Xu J. Gao N. (2019). Long Noncoding RNA MIR17HG Promotes Colorectal Cancer Progression via miR-17-5p. Cancer Res. 79 (19), 4882–4895. 10.1158/0008-5472.can-18-3880 31409641

[B201] Xu J. Shao T. Song M. Xie Y. Zhou J. Yin J. (2020). MIR22HG Acts as a Tumor Suppressor via TGFβ/SMAD Signaling and Facilitates Immunotherapy in Colorectal Cancer. Mol. Cancer 19 (1), 51. 10.1186/s12943-020-01174-w 32127004 PMC7055097

[B202] Xu K. Han B. Bai Y. Ma X.-Y. Ji Z.-N. Xiong Y. (2019). MiR-451a Suppressing BAP31 Can Inhibit Proliferation and Increase Apoptosis through Inducing ER Stress in Colorectal Cancer. Cel Death Dis 10 (3), 152. 10.1038/s41419-019-1403-x PMC6377610 30770794

[B203] Xu K. Zhan Y. Yuan Z. Qiu Y. Wang H. Fan G. (2019). Hypoxia Induces Drug Resistance in Colorectal Cancer through the HIF-1α/miR-338-5p/IL-6 Feedback Loop. Mol. Ther. 27 (10), 1810–1824. 10.1016/j.ymthe.2019.05.017 31208913 PMC6822233

[B204] Xu L. Huan L. Guo T. Wu Y. Liu Y. Wang Q. (2020). LncRNA SNHG11 Facilitates Tumor Metastasis by Interacting with and Stabilizing HIF-1α. Oncogene 39 (46), 7005–7018. 10.1038/s41388-020-01512-8 33060856 PMC7661343

[B205] Xu M. Chen X. Lin K. Zeng K. Liu X. Pan B. (2018). The Long Noncoding RNA SNHG1 Regulates Colorectal Cancer Cell Growth through Interactions with EZH2 and miR-154-5p. Mol. Cancer 17 (1), 141. 10.1186/s12943-018-0894-x 30266084 PMC6162892

[B206] Xu M. Chen X. Lin K. Zeng K. Liu X. Xu X. (2019). lncRNA SNHG6 Regulates EZH2 Expression by Sponging miR-26a/b and miR-214 in Colorectal Cancer. J. Hematol. Oncol. 12 (1), 3. 10.1186/s13045-018-0690-5 30626446 PMC6327409

[B207] Xu M. Xu X. Pan B. Chen X. Lin K. Zeng K. (2019). LncRNA SATB2-AS1 Inhibits Tumor Metastasis and Affects the Tumor Immune Cell Microenvironment in Colorectal Cancer by Regulating SATB2. Mol. Cancer 18 (1), 135. 10.1186/s12943-019-1063-6 31492160 PMC6729021

[B208] Xu X. Huang J. Ocansey D. K. W. Xia Y. Zhao Z. Xu Z. (2021). The Emerging Clinical Application of m6A RNA Modification in Inflammatory Bowel Disease and its Associated Colorectal Cancer. Jir Vol. 14, 3289–3306. 10.2147/jir.s320449 PMC8289367 34290515

[B209] Xu Y. Zhang H. Lit L. C. Grothey A. Athanasiadou M. Kiritsi M. (2014). The Kinase LMTK3 Promotes Invasion in Breast Cancer through GRB2-Mediated Induction of Integrin β₁. Sci. Signal. 7 (330), ra58. 10.1126/scisignal.2005170 24939894

[B210] Xu Y. Zhang H. Nguyen V. T. M. Angelopoulos N. Nunes J. Reid A. (2015). LMTK3 Represses Tumor Suppressor-like Genes through Chromatin Remodeling in Breast Cancer. Cel Rep. 12 (5), 837–849. 10.1016/j.celrep.2015.06.073 26212333

[B211] Xu Z. Zhu C. Chen C. Zong Y. Feng H. Liu D. (2018). CCL19 Suppresses Angiogenesis through Promoting miR-206 and Inhibiting Met/ERK/Elk-1/HIF-1α/VEGF-A Pathway in Colorectal Cancer. Cel Death Dis 9 (10), 974. 10.1038/s41419-018-1010-2 PMC6155262 30250188

[B212] Yan T.-T. Ren L.-L. Shen C.-Q. Wang Z.-H. Yu Y.-N. Liang Q. (2018). miR-508 Defines the Stem-like/Mesenchymal Subtype in Colorectal Cancer. Cancer Res. 78 (7), 1751–1765. 10.1158/0008-5472.can-17-2101 29374066

[B213] Yang M.-H. Zhao L. Wang L. Ou-Yang W. Hu S.-S. Li W.-L. (2019). Nuclear lncRNA HOXD-AS1 Suppresses Colorectal Carcinoma Growth and Metastasis via Inhibiting HOXD3-Induced Integrin β3 Transcriptional Activating and MAPK/AKT Signalling. Mol. Cancer 18 (1), 31. 10.1186/s12943-019-0955-9 30823921 PMC6397497

[B214] Yang P. Li J. Peng C. Tan Y. Chen R. Peng W. (2020). TCONS_00012883 Promotes Proliferation and Metastasis via DDX3/YY1/MMP1/PI3K-AKT axis in Colorectal Cancer. Clin. Transl Med. 10 (6), e211. 10.1002/ctm2.211 33135346 PMC7568852

[B215] Yang X. Zhang S. He C. Xue P. Zhang L. He Z. (2020). METTL14 Suppresses Proliferation and Metastasis of Colorectal Cancer by Down-Regulating Oncogenic Long Non-coding RNA XIST. Mol. Cancer 19 (1), 46. 10.1186/s12943-020-1146-4 32111213 PMC7047419

[B216] Yang Y. Weng W. Peng J. Hong L. Yang L. Toiyama Y. (2017). Fusobacterium Nucleatum Increases Proliferation of Colorectal Cancer Cells and Tumor Development in Mice by Activating Toll-like Receptor 4 Signaling to Nuclear Factor−κB, and Up-Regulating Expression of MicroRNA-21. Gastroenterology 152 (4), 851–866. 10.1053/j.gastro.2016.11.018 27876571 PMC5555435

[B217] Yi H. Geng L. Black A. Talmon G. Berim L. Wang J. (2017). The miR-487b-3p/GRM3/TGFβ Signaling axis Is an Important Regulator of colon Cancer Tumorigenesis. Oncogene 36 (24), 3477–3489. 10.1038/onc.2016.499 28114282 PMC5472494

[B218] Yu M. Luo Y. Cong Z. Mu Y. Qiu Y. Zhong M. (2018). MicroRNA-590-5p Inhibits Intestinal Inflammation by Targeting YAP. J. Crohns Colitis 12 (8), 993–1004. 10.1093/ecco-jcc/jjy046 29912317

[B219] Yu T. Guo F. Yu Y. Sun T. Ma D. Han J. (2017). Fusobacterium Nucleatum Promotes Chemoresistance to Colorectal Cancer by Modulating Autophagy. Cell 170 (3), 548–563. 10.1016/j.cell.2017.07.008 28753429 PMC5767127

[B220] Yu T. Shan T.-D. Li J.-Y. Huang C.-Z. Wang S.-Y. Ouyang H. (2016). Knockdown of Linc-UFC1 Suppresses Proliferation and Induces Apoptosis of Colorectal Cancer. Cel Death Dis 7, e2228. 10.1038/cddis.2016.124 PMC4917661 27195675

[B221] Yu X. Yuan Z. Yang Z. Chen D. Kim T. Cui Y. (2018). The Novel Long Noncoding RNA U50535 Promotes Colorectal Cancer Growth and Metastasis by Regulating CCL20. Cel Death Dis 9 (7), 751. 10.1038/s41419-018-0771-y PMC6030363 29970882

[B222] Yu Y. Nangia-Makker P. Farhana L. Majumdar A. P. N. (2017). A Novel Mechanism of lncRNA and miRNA Interaction: CCAT2 Regulates miR-145 Expression by Suppressing its Maturation Process in colon Cancer Cells. Mol. Cancer 16 (1), 155. 10.1186/s12943-017-0725-5 28964256 PMC5622467

[B223] Yue B. Cai D. Liu C. Fang C. Yan D. (2016). Linc00152 Functions as a Competing Endogenous RNA to Confer Oxaliplatin Resistance and Holds Prognostic Values in Colon Cancer. Mol. Ther. 24 (12), 2064–2077. 10.1038/mt.2016.180 27633443 PMC5167786

[B224] Yue B. Liu C. Sun H. Liu M. Song C. Cui R. (2018). A Positive Feed-Forward Loop between LncRNA-CYTOR and Wnt/β-Catenin Signaling Promotes Metastasis of Colon Cancer. Mol. Ther. 26 (5), 1287–1298. 10.1016/j.ymthe.2018.02.024 29606502 PMC5993983

[B225] Yue C. Chen J. Li Z. Li L. Chen J. Guo Y. (2020). microRNA-96 Promotes Occurrence and Progression of Colorectal Cancer via Regulation of the AMPKα2-FTO-m6A/MYC axis. J. Exp. Clin. Cancer Res. 39 (1), 240. 10.1186/s13046-020-01731-7 33183350 PMC7659164

[B226] Zeng K. Chen X. Xu M. Liu X. Hu X. Xu T. (2018). CircHIPK3 Promotes Colorectal Cancer Growth and Metastasis by Sponging miR-7. Cel Death Dis 9 (4), 417. 10.1038/s41419-018-0454-8 PMC5856798 29549306

[B227] Zeng Z. Li Y. Pan Y. Lan X. Song F. Sun J. (2018). Cancer-derived Exosomal miR-25-3p Promotes Pre-metastatic Niche Formation by Inducing Vascular Permeability and Angiogenesis. Nat. Commun. 9 (1), 5395. 10.1038/s41467-018-07810-w 30568162 PMC6300604

[B228] Zhan W. Liao X. Chen Z. Li L. Tian T. Yu L. (2020). LINC00858 Promotes Colorectal Cancer by Sponging miR-4766-5p to Regulate PAK2. Cel Biol Toxicol 36 (4), 333–347. 10.1007/s10565-019-09506-3 31902050

[B229] Zhang F. Luo Y. Shao Z. Xu L. Liu X. Niu Y. (2016). MicroRNA-187, a Downstream Effector of TGFβ Pathway, Suppresses Smad-Mediated Epithelial-Mesenchymal Transition in Colorectal Cancer. Cancer Lett. 373 (2), 203–213. 10.1016/j.canlet.2016.01.037 26820227

[B230] Zhang M. Weng W. Zhang Q. Wu Y. Ni S. Tan C. (2018). The lncRNA NEAT1 Activates Wnt/β-Catenin Signaling and Promotes Colorectal Cancer Progression via Interacting with DDX5. J. Hematol. Oncol. 11 (1), 113. 10.1186/s13045-018-0656-7 30185232 PMC6125951

[B231] Zhang Y. Guo L. Li Y. Feng G.-H. Teng F. Li W. (2018). MicroRNA-494 Promotes Cancer Progression and Targets Adenomatous Polyposis Coli in Colorectal Cancer. Mol. Cancer 17 (1), 1. 10.1186/s12943-017-0753-1 29304823 PMC5755155

[B232] Zhang Z. Zhou C. Chang Y. Zhang Z. Hu Y. Zhang F. (2016). Long Non-coding RNA CASC11 Interacts with hnRNP-K and Activates the WNT/β-catenin Pathway to Promote Growth and Metastasis in Colorectal Cancer. Cancer Lett. 376 (1), 62–73. 10.1016/j.canlet.2016.03.022 27012187

[B233] Zhao B. Wei X. Li W. Udan R. S. Yang Q. Kim J. (2007). Inactivation of YAP Oncoprotein by the Hippo Pathway Is Involved in Cell Contact Inhibition and Tissue Growth Control. Genes Dev. 21 (21), 2747–2761. 10.1101/gad.1602907 17974916 PMC2045129

[B234] Zhao S. Sun H. Jiang W. Mi Y. Zhang D. Wen Y. (2017). miR-4775 Promotes Colorectal Cancer Invasion and Metastasis via the Smad7/TGFβ-Mediated Epithelial to Mesenchymal Transition. Mol. Cancer 16 (1), 12. 10.1186/s12943-017-0585-z 28095858 PMC5240405

[B235] Zhao Y. Du T. Du L. Li P. Li J. Duan W. (2019). Long Noncoding RNA LINC02418 Regulates MELK Expression by Acting as a ceRNA and May Serve as a Diagnostic Marker for Colorectal Cancer. Cel Death Dis 10 (8), 568. 10.1038/s41419-019-1804-x PMC6662768 31358735

[B236] Zheng K. Zhou X. Yu J. Li Q. Wang H. Li M. (2016). Epigenetic Silencing of miR-490-3p Promotes Development of an Aggressive Colorectal Cancer Phenotype through Activation of the Wnt/β-Catenin Signaling Pathway. Cancer Lett. 376 (1), 178–187. 10.1016/j.canlet.2016.03.024 27037061

[B237] Zhou Q. Zhu Y. Wei X. Zhou J. Chang L. Sui H. (2016). MiR-590-5p Inhibits Colorectal Cancer Angiogenesis and Metastasis by Regulating Nuclear Factor 90/vascular Endothelial Growth Factor A axis. Cel Death Dis 7 (10), e2413. 10.1038/cddis.2016.306 PMC5133975 27735951

[B238] Zhou T. Wu L. Ma N. Tang F. Yu Z. Jiang Z. (2020). SOX9-activated FARSA-AS1 Predetermines Cell Growth, Stemness, and Metastasis in Colorectal Cancer through Upregulating FARSA and SOX9. Cel Death Dis 11 (12), 1071. 10.1038/s41419-020-03273-4 PMC7736271 33318478

[B239] Zhu G.-f. Xu Y.-w. Li J. Niu H.-l. Ma W.-x. Xu J. (2019). Mir20a/106a-WTX axis Regulates RhoGDIa/CDC42 Signaling and colon Cancer Progression. Nat. Commun. 10 (1), 112. 10.1038/s41467-018-07998-x 30631060 PMC6328557

[B240] Zhu P. Wu J. Wang Y. Zhu X. Lu T. Liu B. (2018). LncGata6 Maintains Stemness of Intestinal Stem Cells and Promotes Intestinal Tumorigenesis. Nat. Cel Biol 20 (10), 1134–1144. 10.1038/s41556-018-0194-0 30224759

[B241] Zhu Y. Gu L. Li Y. Lin X. Shen H. Cui K. (2017). miR-148a Inhibits Colitis and Colitis-Associated Tumorigenesis in Mice. Cel Death Differ 24 (12), 2199–2209. 10.1038/cdd.2017.151 PMC5686357 28960206

[B242] Zhu Y. Gu L. Lin X. Cui K. Liu C. Lu B. (2020). LINC00265 Promotes Colorectal Tumorigenesis via ZMIZ2 and USP7-Mediated Stabilization of β-catenin. Cel Death Differ 27 (4), 1316–1327. 10.1038/s41418-019-0417-3 PMC7206056 31527801

[B243] Zhu Y. Hu H. Yuan Z. Zhang Q. Xiong H. Hu Z. (2020). LncRNA NEAT1 Remodels Chromatin to Promote the 5-Fu Resistance by Maintaining Colorectal Cancer Stemness. Cel Death Dis 11 (11), 962. 10.1038/s41419-020-03164-8 PMC7653927 33168814

[B244] Zhu Y. Wang C. Becker S. A. Hurst K. Nogueira L. M. Findlay V. J. (2018). miR-145 Antagonizes SNAI1-Mediated Stemness and Radiation Resistance in Colorectal Cancer. Mol. Ther. 26 (3), 744–754. 10.1016/j.ymthe.2017.12.023 29475734 PMC5910672

